# GIRK2 potassium channels expressed by the AgRP neurons decrease adiposity and body weight in mice

**DOI:** 10.1371/journal.pbio.3002252

**Published:** 2023-08-18

**Authors:** Youjin Oh, Eun-Seon Yoo, Sang Hyeon Ju, Eunha Kim, Seulgi Lee, Seyun Kim, Kevin Wickman, Jong-Woo Sohn

**Affiliations:** 1 Department of Biological Sciences, Korea Advanced Institute of Science and Technology, Daejeon, South Korea; 2 Department of Internal Medicine, Chungnam National University Hospital, Daejeon, South Korea; 3 Department of Pharmacology, University of Minnesota, Minneapolis, Minnesota, United States of America; INSERM, FRANCE

## Abstract

It is well known that the neuropeptide Y (NPY)/agouti-related peptide (AgRP) neurons increase appetite and decrease thermogenesis. Previous studies demonstrated that optogenetic and/or chemogenetic manipulations of NPY/AgRP neuronal activity alter food intake and/or energy expenditure (EE). However, little is known about intrinsic molecules regulating NPY/AgRP neuronal excitability to affect long-term metabolic function. Here, we found that the G protein-gated inwardly rectifying K^+^ (GIRK) channels are key to stabilize NPY/AgRP neurons and that NPY/AgRP neuron-selective deletion of the GIRK2 subunit results in a persistently increased excitability of the NPY/AgRP neurons. Interestingly, increased body weight and adiposity observed in the NPY/AgRP neuron-selective GIRK2 knockout mice were due to decreased sympathetic activity and EE, while food intake remained unchanged. The conditional knockout mice also showed compromised adaptation to coldness. In summary, our study identified GIRK2 as a key determinant of NPY/AgRP neuronal excitability and driver of EE in physiological and stress conditions.

## Abstract 2

It is known that a subset of neurons in the hypothalamus increase appetite and decrease thermogenesis. This study shows that the ion channel GIRK2 functions in hypothalamic NPY/AgRP neurons to increase energy expenditure in mice; activity of this ion channel may help to keep body weight under control.

## Introduction

The arcuate nucleus of the hypothalamus (ARH) is home to several distinct types of neurons that control energy homeostasis [1]. In particular, it is well known that neurons co-expressing neuropeptide Y (NPY) and agouti-related peptide (AgRP) (NPY/AgRP neurons) promote food intake [2,3]. NPY/AgRP neurons also decrease energy expenditure (EE), at least in part by suppressing sympathetic tone to the brown adipose tissue (BAT) and inhibiting thermogenesis [4,5]. Consistent with these findings, manipulating the activity of NPY/AgRP neurons using exogenous genetic constructs (e.g., channelrhodopsin and designer receptors) resulted in acute changes in food intake and energy utilization [6,7]. While these studies provided insight into how NPY/AgRP neuronal activity is translated to in vivo metabolic function, we have little information regarding intrinsic molecules that regulate NPY/AgRP neuronal activity per se.

In many excitable cells, the resting membrane potential (RMP) is maintained largely by K^+^ channels [8]. For example, the “classic” inwardly rectifying K^+^ (IRK or Kir2) channels maintain RMP of cardiac myocytes [9] and ATP-sensitive K^+^ (K_ATP_) channels silence pancreatic β-cells [10]. In neurons, K_ATP_ channels and G protein-gated inwardly rectifying K^+^ (GIRK or Kir3) channels have been reported to open at rest to dampen cellular excitability. For example, K_ATP_ channel activity hyperpolarizes membrane potential of the pro-opiomelanocortin (POMC) neurons of the ARH [11] and the serotonin 2C receptor-expressing neurons of the lateral parabrachial nucleus [12]. It was also demonstrated that GIRK channels maintain RMP of arcuate POMC neurons [13] and hippocampal CA1 neurons [14]. However, little data is currently available on the identity of K^+^ channels that regulate RMP of NPY/AgRP neurons.

In this study, we utilized multiple approaches to identify specific K^+^ channels that regulate NPY/AgRP neuronal activity. Firstly, we found evidence that GIRK2-containing GIRK channels suppress the activity of NPY/AgRP neurons. We subsequently found that GIRK2 ablation in NPY/AgRP neurons results in increased body weight and adiposity when the mice are fed normal chow diet (NCD). Notably, the observed phenotypes were attributed to decreased sympathetic activity and energy expenditure, rather than an increase of food intake. We also found evidence that GIRK2 expressed by NPY/AgRP neurons has a role in cold-induced thermogenesis. Collectively, our results suggest that GIRK2 dampens excitability of the NPY/AgRP neurons to maintain sympathetic tone and thermogenesis in physiological and some stress conditions, which may serve to keep body weight in control independently of appetite.

## Results

### GIRK channels maintain RMP of NPY neurons

**Fig 1:**
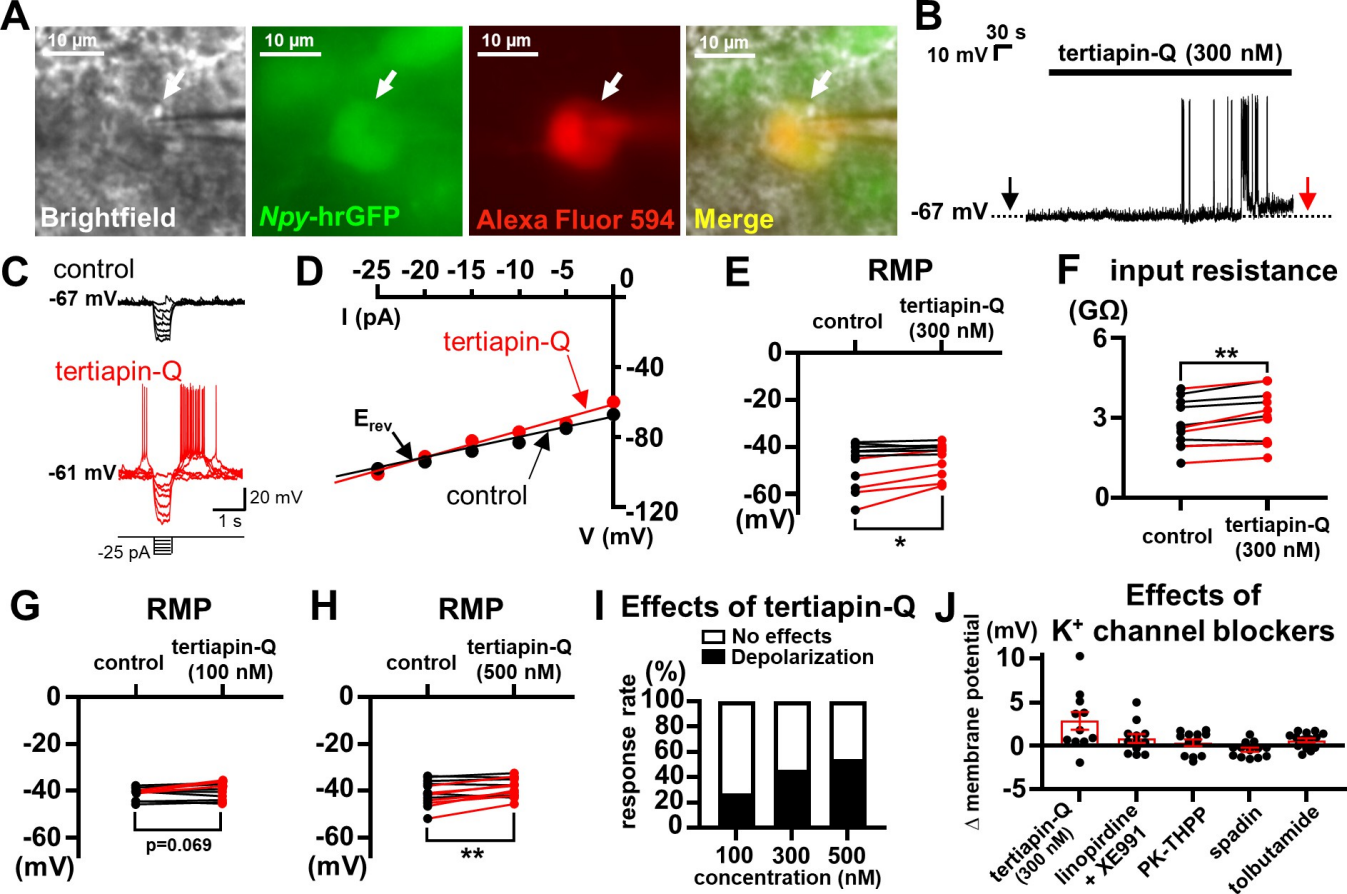
GIRK channels stabilize RMP of NPY neurons. (A) Brightfield illumination (Brightfield), fluorescent (FITC) illumination (*Npy*-hrGFP), fluorescent (TRITC) illumination (Alexa Fluor 594), and merged (Merge) images of targeted NPY neuron. Arrows indicate the cell targeted for whole-cell patch clamp recording. (B) Image demonstrates a depolarizing effect of tertiapin-Q. Dotted line indicates RMP. (C) Voltage deflections in response to small hyperpolarizing current steps (from −25 pA to 0 pA by 5 pA increments) before (control, black) and after (tertiapin-Q, red) the perfusion with tertiapin-Q as indicated by arrows in (B). (D) The voltage–current (V-I) relationship demonstrates increased input resistance by tertiapin-Q. E_rev_ = reversal potential. (E) Lines and dots summarize effects of tertiapin-Q on RMP (from −47.7 ± 3.0 mV to −44.9 ± 2.1 mV, *n* = 11, df = 10, t = 2.787, *p* = 0.019). Red and black lines indicate changes of membrane potential in depolarized and nonresponsive neurons, respectively. (F) Lines and dots summarize effect of tertiapin-Q on input resistance (from 2.75 ± 0.27 GΩ to 3.03 ± 0.30 GΩ, *n* = 11, df = 10, t = 4.370, *p* = 0.001). Red and black lines indicate changes of input resistance in depolarized and nonresponsive neurons, respectively. (G, H) Lines and dots summarize effects of 100 nM tertiapin-Q (G) (from −41.2 ± 0.8 mV to −40.0 ± 1.1 mV, *n* = 11, df = 10, t = 2.040, *p* = 0.069) and 500 nM tertiapin-Q (H) (from −42.9 ± 1.2 mV to −40.5 ± 1.1 mV, *n* = 13, df = 12, t = 3.292, *p* = 0.006) on RMP. Red and black lines indicate changes of membrane potential in depolarized and nonresponsive neurons, respectively. (I) Histogram summarizes responses (no effects or depolarization) of NPY neurons to different concentrations of tertiapin-Q. (J) Bar graphs and dots summarize effects of K^+^ channel blockers. Each neuron was tested with only 1 K^+^ channel blocker. Data are presented as mean ± SEM. Paired *t* test was used for statistical analyses. *p < 0.05, **p < 0.01. The numerical data for Fig 1D–1J can be found in S1 Data. GIRK, G protein-gated inwardly rectifying K^+^; NPY, neuropeptide Y; RMP, resting membrane potential.

A previous study reported a transcriptome obtained from AgRP neurons [15], which included mRNA of various K^+^ channels that may contribute to maintenance of RMP. In particular, M-type K^+^ or M channels, two-pore K^+^ (K2P) channels, K_ATP_ channels, and GIRK channels had significant levels of mRNA expression [15]. Thus, we obtained acute hypothalamic slices from the *Npy*-hrGFP mice and targeted the fluorescence-labeled NPY neurons within the ARH for whole-cell patch clamp recordings (Fig 1A), where we tested the effects of pharmacological inhibitors of abovementioned K^+^ channels.

GIRK channels were demonstrated to maintain RMP of several types of central neurons [13,16,17]. We tested the involvement of GIRK channels and found that bath applications of tertiapin-Q (300 nM), a GIRK channel blocker, depolarized membrane potential in 5 of 11 (approximately 45%) NPY neurons (from −56.3 ± 3.6 mV to −50.6 ± 2.8 mV, *n* = 5, Fig 1B and 1E, red lines). We applied small hyperpolarizing current steps before and after tertiapin-Q treatments (Fig 1B and 1C) and plotted the amplitudes of voltage responses against the amplitudes of injected currents to obtain a voltage–current (V-I) relationship (Fig 1D). We noted that the depolarizing effects were accompanied by increased input resistance (from 2.49 ± 0.47 GΩ to 2.84 ± 0.50 GΩ, *n* = 5, Fig 1F, red lines) with a reversal potential (E_rev_) of −101.3 ± 14.6 mV (*n* = 5) (Fig 1D). Changes of membrane potential and input resistance by tertiapin-Q were significant when we included all neurons recorded (Fig 1E and 1F). We also tested lower (100 nM) and higher (500 nM) concentrations of tertiapin-Q and found that the depolarizing effects become more significant at higher concentrations (Fig 1G and 1H). In addition, the response rate increased at higher concentrations (Fig 1I). These results suggested the contribution of GIRK channels to the maintenance of RMP in NPY neurons.

Notably, NPY neurons depolarized by tertiapin-Q had significantly lower action potential (AP) firing frequency and hyperpolarized RMP compared to those not responding to tertiapin-Q (S1A and S1B Fig). These data suggest that NPY neurons depolarized by tertiapin-Q have active GIRK channels and therefore are more stable. Consistent with this idea, NPY neurons depolarized by tertiapin-Q had lower input resistance than nonresponsive neurons, although the difference was not significant (S1C Fig). We also noted lower AP threshold in neurons depolarized by tertiapin-Q (S1D Fig), which may result from higher availability of voltage-gated Na^+^ channels due to more negative RMP.

Subsequently, we tested the effects of M channel blockers (10 μM linopirdine and 10 μM XE991) and observed depolarizing responses (3 mV and 5 mV) in 2 of 12 cells (approximately 17%) tested (Figs 1J, S2A, and S2E). These effects were accompanied by increased input resistance (from 2.98 GΩ to 3.80 GΩ and from 3.56 GΩ to 4.84 GΩ) and E_rev_ of −94.0 mV and −81.0 mV, which suggested the contribution of M channels in a small subpopulation of NPY neurons. We also tested the effects of PK-THPP (1 μM, a TASK-3 channel blocker), spadin (1 μM, a TREK-1 channel blocker), and tolbutamide (100 μM, a K_ATP_ channel blocker), but none of these blockers caused significant changes in NPY neuronal membrane potential (Figs 1J, S2B–S2D, and S2F–S2H). Since we included 2 mM of ATP in pipette solutions (see Materials and methods), which may inhibit K_ATP_ channels [18], we also tested the effects of tolbutamide using ATP-free pipette solutions but found that RMP still remains unchanged (from −41.1 ± 1.1 mV to −40.9 ± 1.1 mV, *p* = 0.623, *n* = 10). Thus, it appears that neither K2P channel nor K_ATP_ channel plays a measurable role to maintain RMP of NPY neurons.

### Arcuate AgRP neurons preferentially express *Girk2* over *Girk1*

**Fig 2:**
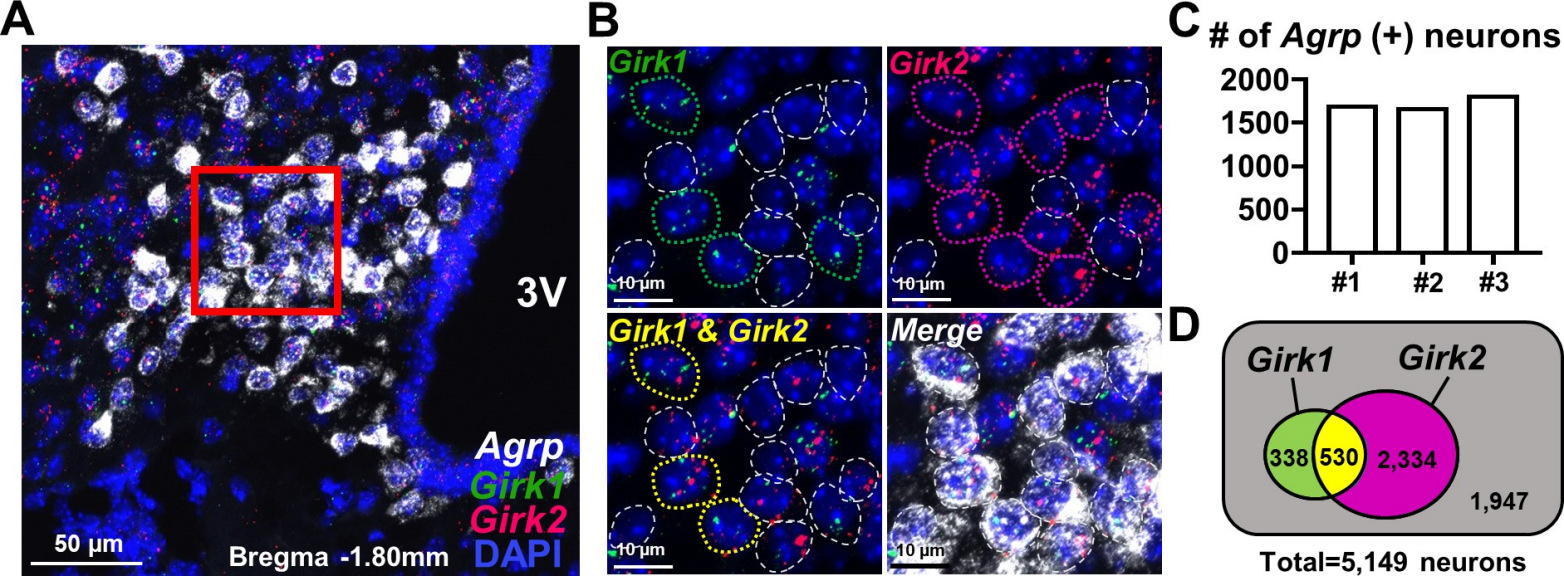
Dominant expression of *Girk2* over *Girk1* by the arcuate AgRP neurons. (A) Image demonstrates DAPI (blue) and mRNA of *Agrp* (white), *Girk1* (green), and *Girk2* (magenta) detected by FISH experiments within the arcuate nucleus. 3V = third ventricle. Scale bar = 50 μm. (B) Magnified images of red rectangular area in (A). Dotted circles indicate *Agrp* (+) neurons (white) with *Girk1* (green), *Girk2* (magenta), or both *Girk1* and *Girk2* (yellow) mRNA. Scale bar = 10 μm. (C) Bar graph demonstrates numbers of *Agrp* (+) neurons in the arcuate nuclei of 3 wild-type mice. (D) Venn diagram demonstrates the numbers of *Girk1*- and/or *Girk2*-expressing *Agrp* (+) neurons. Data were pooled from neurons of 3 mice shown in (C), and 12 hypothalamic slices from each mouse (from bregma −1.58 mm to −2.02 mm) were included for analyses. The numerical data for Fig 2C can be found in S2 Data. AgRP, agouti-related peptide; FISH, fluorescence in situ hybridization; GIRK, G protein-gated inwardly rectifying K^+^.

Neuronal GIRK channels contain one or both of GIRK1 and GIRK2 subunits [19], and both *Girk1* and *Girk2* mRNAs were found in the transcriptome of AgRP neurons [15]. Therefore, we characterized the expression of *Girk1* and *Girk2* by arcuate AgRP neurons with fluorescence in situ hybridization (FISH) experiments (RNAscope) targeting *Agrp*, *Girk1*, and *Girk2* mRNA in wild-type mice. As shown in Fig 2A and 2B, *Agrp*-expressing neurons (white) expressed both *Girk1* (green) and *Girk2* (magenta) at mRNA levels within the ARH.

For quantitative analyses, we pooled 5,149 *Agrp*-positive ARH neurons from 3 mice, each of which had comparable numbers of *Agrp*-expressing neurons in coronal hypothalamic sections from bregma −1.58 mm to −2.02 mm (Fig 2C). We found that 3,202 neurons (62.2%) express either *Girk1* or *Girk2* mRNA (Fig 2D), where *Girk2* is expressed by a majority (2,864 neurons, 55.6%) but *Girk1* by a smaller subpopulation (868 neurons, 16.9%). Moreover, almost two thirds of *Girk1*-expressing neurons also expressed *Girk2* (530 neurons, 10.3%), whereas 338 neurons (6.6%) expressed *Girk1* only. The remaining 1,947 *Agrp*-positive neurons (37.8%) did not express either *Girk1* or *Girk2*. We also noted rather even distribution of both *Girk1* and *Girk2* along the rostrocaudal axis of the hypothalamus containing *Agrp*-positive neurons (S3A Fig). These results demonstrate that the arcuate AgRP neurons express both *Girk1* and *Girk2*, but the latter is expressed by a larger subpopulation.

We repeated the same series of experiments for all possible combinations of functional GIRK channels and confirmed that *Girk2* expression is higher than any other subunit (S3B–S3D Fig). The *Girk2* mRNA was expressed by 55.8 ± 1.7% (*n* = 3) and 50.9 ± 1.5% (*n* = 3) of *Agrp*-positive neurons in 2 independent sets of experiments (S3A and S3D Fig). Expression levels of *Girk3* and *Girk4* mRNA were comparable to each other: *Girk3* mRNA was expressed by 34.5 ± 1.7% (*n* = 3) and 29.2 ± 1.9% (*n* = 3) of *Agrp*-positive neurons in 2 independent sets of experiments (S3B and S3D Fig), while *Girk4* mRNA was expressed by 30.1 ± 1.0% (*n* = 3) of *Agrp*-positive neurons in 1 set of experiments (S3C Fig). *Girk1* mRNA showed the least abundant expression and was expressed by 16.8 ± 0.9% (*n* = 3), 17.7 ± 1.2% (*n* = 3) and 16.1 ± 0.5% (*n* = 3) of *Agrp*-positive neurons in 3 independent sets of experiments (S3A–S3C Fig). Notably, co-expression of any 2 subunits (i.e., *Girk1*/*Girk2*, *Girk1*/*Girk3*, *Girk1*/*Girk4*, *Girk2*/*Girk3*, gray lines in S3A–S3D Fig) was found in a minority of *Agrp*-positive neuron. The highest level of co-expression was observed for *Girk2* and *Girk3*: These subunits are co-expressed by 18.3 ± 1.4% (*n* = 3) of *Agrp*-positive neurons (S3D Fig), while the lowest level of co-expression was observed for *Girk1* and *Girk3* (co-expression by 7.7 ± 0.8% (*n* = 3) of *Agrp*-positive neurons (S3B Fig)). *Girk1*/*Girk2* co-expression and *Girk1*/*Girk4* co-expression was observed in 10.2 ± 0.7% (*n* = 3) and 8.1 ± 0.6% (*n* = 3) of *Agrp*-positive neurons, respectively (S3A and S3C Fig). These results suggest that GIRK2 homomers, followed by GIRK2/GIRK3 heteromers, constitute a majority of GIRK channels in NPY/AgRP neurons.

### GIRK2-containing GIRK channels are dispensable for GABA_B_-activated K^+^ currents in NPY neurons

GIRK channels are known to mediate slow synaptic inhibition by the stimulation of GABA_B_ receptors [20]. Thus, we performed voltage clamp experiments to determine whether GIRK channels contribute to GABA_B_-activated currents in NPY neurons. We applied baclofen, a GABA_B_ receptor agonist, to NPY neurons from the *Npy*-hrGFP transgenic mice using a local perfusion system (see Materials and methods) to record GABA_B_-activated GIRK currents. At a holding potential of −40 mV, application of 100 μM baclofen caused instantaneous outward currents (S4A Fig). We applied voltage ramp pulses (from −120 mV to −10 mV, 100 mV/s) before and during baclofen applications (arrows “a” and “b” of S4A Fig) to obtain a current–voltage (I-V) relationship of baclofen-activated currents (*I*_Bac_), where *I*_Bac_ was *I*_b_-*I*_a_ (S4B Fig). The I-V relationship of *I*_Bac_ showed inward rectification with E_rev_ close to E_K_ (−88.5 ± 0.7 mV, *n* = 12), consistent with GIRK channel activation. We also calculated the rectification index (*I*_-120 mV_/*I*_-60 mV_), the ratio of absolute values of currents at −120 mV (*I*_-120 mV_) and −60 mV (*I*_-60 mV_) of I-V curve. The average rectification index was 2.5 ± 0.2 (*n* = 12, S4C Fig).

We next examined currents evoked by baclofen (10 μM and 100 μM) in NPY neurons on WT (NPY^G2WT^ neuron) and GIRK2 KO (NPY^G2KO^ neuron) backgrounds (see Materials and methods). Unexpectedly, we found that GIRK2 ablation did not affect the amplitudes of *I*_Bac_ at 10 μM (1.4 ± 0.1 pA/pF, *n* = 32, for NPY^G2WT^ neuron and 1.4 ± 0.1 pA/pF, *n* = 23, for NPY^G2KO^ neuron, *p* = 0.783) and at 100 μM (1.8 ± 0.1 pA/pF, *n* = 53, for NPY^G2WT^ neuron and 1.8 ± 0.2 pA/pF, *n* = 26, for NPY^G2KO^ neuron, *p* = 0.984), respectively (S4D–S4G Fig). These results demonstrate that GIRK2-containing GIRK channels are not responsible for GABA_B_-activated K^+^ currents in NPY neurons.

### GIRK2-containing GIRK channels contribute to RMP of NPY neurons

**Fig 3:**
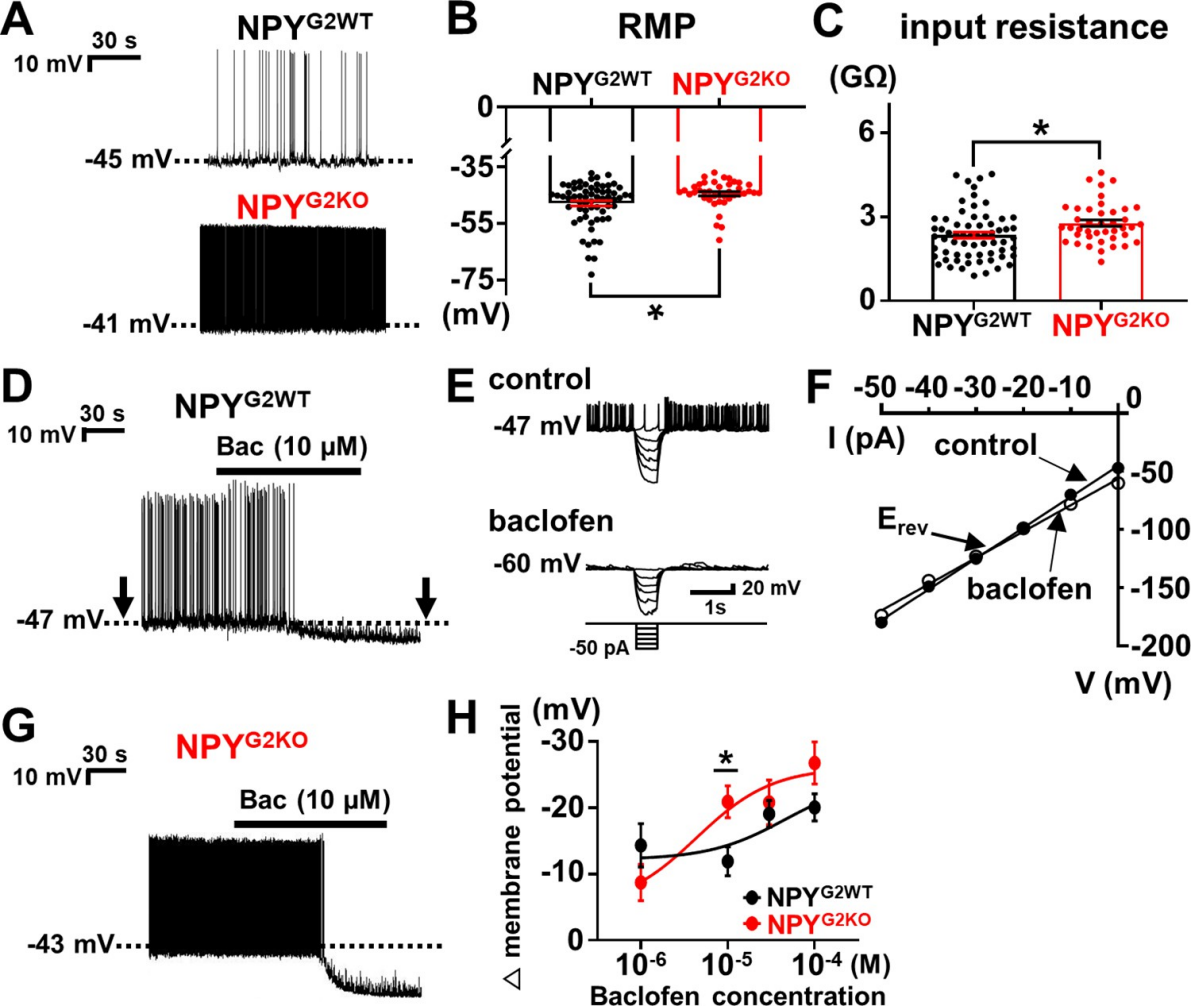
Contribution of GIRK2-containing channels to RMP and GABA_B_-induced inhibition of NPY neurons. (A) Traces demonstrate spontaneous firing and RMP of NPY^G2WT^ (black) and NPY^G2KO^ (red) neurons. Dotted line indicates RMP. (B, C) Bar graphs and dots summarize RMP (−47.9 ± 0.9 mV, *n* = 64, for NPY^G2WT^ and −44.5 ± 0.7 mV, *n* = 41, for NPY^G2KO^, df = 103, t = 2.556, *p* = 0.012) (B) and input resistance (2.35 ± 0.11 GΩ, *n* = 64, for NPY^G2WT^ and 2.78 ± 0.11 GΩ, *n* = 41, for NPY^G2KO^, df = 103, t = 2.590, *p* = 0.011) (C) of NPY^G2WT^ (*n* = 64, black) and NPY^G2KO^ (*n* = 41, red) neurons. (D) Image demonstrates a hyperpolarization of NPY^G2WT^ neuron membrane potential by baclofen (10 μM). Arrows indicate interruptions to apply current step pulses. (E) Small hyperpolarizing current steps (from −50 pA to 0 pA by 10 pA increments) were applied before (control) and after (baclofen) applications of baclofen. (F) Voltage–current relationship demonstrates decreased input resistance and E_rev_ close to E_K_. (G) Image demonstrates a hyperpolarization of NPY^G2KO^ neuron membrane potential by baclofen (10 μM). (H) Summary of GABA_B_-induced hyperpolarization of NPY^G2WT^ (black) and NPY^G2KO^ (red) neurons. Changes of membrane potential by 10 μM baclofen was −11.9 ± 2.2 mV for NPY^G2WT^ (*n* = 14) and −20.9 ± 2.4 mV for NPY^G2KO^ (*n* = 8) (df = 20, t = 2.655, *p* = 0.015). Solid lines indicate fitting of dose-response curve (Hill slope = 1.0, Y = Bottom + (Top-Bottom)/(1+10^(logEC_50_-X)). Both hyperpolarizing and no responses were included for analyses. See Table 1 for hyperpolarizing responses only. Data are presented as mean ± SEM. Unpaired *t* test was used for statistical analyses. *p < 0.05. The numerical data for Fig 3B, 3C, 3F, and 3H can be found in S3 Data. GIRK, G protein-gated inwardly rectifying K^+^; NPY, neuropeptide Y; RMP, resting membrane potential.

We also performed current clamp experiments to assess the role of GIRK2-containing GIRK channels in the maintenance of NPY neuron RMP. We found that NPY^G2KO^ neurons had significantly depolarized RMP (−44.5 ± 0.7 mV, *n* = 41, *p* = 0.012) compared to NPY^G2WT^ neurons (−47.9 ± 0.9 mV, *n* = 64) (Fig 3A and 3B). NPY^G2KO^ neurons also had significantly higher input resistance (2.78 ± 0.11 GΩ, *n* = 41, *p* = 0.011) compared to NPY^G2WT^ neurons (2.35 ± 0.11 GΩ, *n* = 64) (Fig 3C). In addition, tertiapin-Q (300 nM) depolarized only 1 of 13 (7.7%) NPY^G2KO^ neurons, which was significantly different from the effects of tertiapin-Q on NPY^G2WT^ neurons (S5 Fig). Together with results from S1 and S3 Figs, it seems that GIRK2-containing GIRK channels are open at rest to maintain RMP and decrease input resistance in NPY neurons.

We also examined whether GIRK2-containing GIRK channels have a role in GABA_B_-induced hyperpolarization of NPY neuronal membrane potential. We noted that treatments of NPY^G2WT^ neurons with CGP54626 (2 μM), a GABA_B_ receptor antagonist, do not affect RMP and input resistance of NPY^G2WT^ neurons (S6 Fig), which suggested that GABA_B_ receptors are not active at rest to affect the membrane potential of NPY^G2WT^ neurons. Subsequently, we found that application of 10 μM baclofen hyperpolarized NPY^G2WT^ neurons by −14.1 ± 1.9 mV (*n* = 12 of 14 cells) (Fig 3D and Table 1). The hyperpolarizing effects were accompanied by decreased input resistance and E_rev_ of −104.3 ± 5.3 mV (*n* = 12) based on the V-I relationship calculated from voltage responses to current steps pulses before and after baclofen perfusion (Fig 3E and 3F). We also tried lower (1 μM) and higher (30 μM and 100 μM) concentrations of baclofen and noted dose-dependent effects (Fig 3H and Table 1), where E_rev_ was comparable across all concentrations tested. We conducted the same series of experiments with NPY^G2KO^ neurons and found that NPY^G2KO^ neurons showed significantly augmented hyperpolarization (−20.9 ± 2.4 mV, *n* = 8, *p* = 0.035) by 10 μM baclofen (Fig 3G and 3H and Table 1). The observed augmentation of baclofen-induced hyperpolarization is likely due to increased input resistance together with unchanged GABA_B_-activated GIRK currents in NPY^G2KO^ neurons (S4D–S4G Fig).

**Table 1:** Summary of GABA_B_-induced hyperpolarization of arcuate NPY neurons.

Baclofen Conc.	NPY^G2WT^ neuron	NPY^G2KO^ neuron
1 μM	−16.3 ± 3.0 mV (*n* = 7, 88%) E_rev_ = −94.9 ± 6.1 mV	−13.1 ± 2.4 mV (*n* = 6, 67%, *p* = 0.431) E_rev_ = −99.8 ± 11.1 mV
10 μM	−14.1 ± 1.9 mV (*n* = 12, 86%) E_rev_ = −104.3 ± 5.3 mV	−20.9 ± 2.4 mV (*n* = 8, 100%, *p* = 0.035) E_rev_ = −94.3 ± 2.0 mV
30 μM	−19.1 ± 2.0 mV (*n* = 12, 100%) E_rev_ = −99.6 ± 4.8 mV	−20.8 ± 3.3 mV (*n* = 9, 100%, *p* = 0.641) E_rev_ = −83.1 ± 3.8 mV
100 μM	−20.0 ± 2.0 mV (*n* = 14, 100%) E_rev_ = −92.7 ± 3.1 mV	−26.7 ± 3.2 mV (*n* = 9, 100%, *p* = 0.075) E_rev_ = −89.8 ± 3.7 mV

Changes of membrane potential are presented as mean ± SEM. Numbers in parentheses indicate the number of responsive cells, response rate, and *p* values (unpaired *t* test, NPY^G2WT^ neurons vs. NPY^G2KO^ neurons). E_rev_ = reversal potential. The individual numerical data for changes of membrane potential and reversal potential can be found in S8 Data.

NPY, neuropeptide Y.

### GIRK2 ablation, but not GIRK1 ablation, results in a persistent increase of AgRP neuronal activity

**Fig 4:**
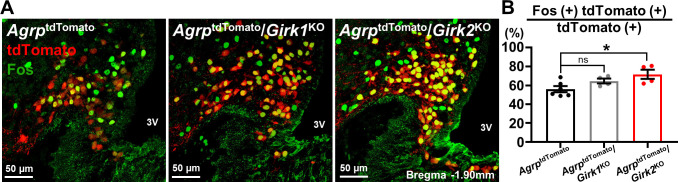
Deletion of GIRK2, but not GIRK1, leads to increased Fos expression by the arcuate AgRP neurons. (A) Images demonstrate Fos IHC results from *Agrp*^tdTomato^, *Agrp*^tdTomato^/*Girk1*^KO^, and *Agrp*^tdTomato^/*Girk2*^KO^ mice, as indicated. 3V = third ventricle. Scale bar = 50 μm. (B) Bar graphs and dots summarize proportion of Fos-expressing AgRP neurons in *Agrp*^tdTomato^ (56.0 ± 3.2%, *n* = 6, black), *Agrp*^tdTomato^/*Girk1*^KO^ (64.6 ± 2.6%, *n* = 4, gray), and *Agrp*^tdTomato^/*Girk2*^KO^ (71.7 ± 4.8%, *n* = 4, red). Twelve hypothalamic slices from each mouse (from bregma −1.46 mm to −2.06 mm) were included for analyses. Data are presented as mean ± SEM. Ordinary one-way ANOVA with Bonferroni correction was used for statistical analyses (df = 2, *F*_*2*, *11*_ = 4.961, *p* = 0.029). *p < 0.05, ns = not significant. The numerical data for Fig 4B can be found in S4 Data. AgRP, agouti-related peptide; GIRK, G protein-gated inwardly rectifying K^+^; IHC, immunohistochemistry.

Given the higher expression of *Girk2* mRNA than *Girk1* mRNA (Fig 2) as well as the contribution of GIRK2 subunits to the RMP (Fig 3), we assumed that the GIRK2-containing GIRK channels may play a more important role than GIRK1-containing GIRK channels to maintain AgRP neuronal activity. To test this idea, we labeled AgRP neurons with tdTomato reporter using *Agrp*-ires-Cre::tdTomato (*Agrp*^tdTomato^) mice and performed immunohistochemistry (IHC) experiments to measure Fos expression level in arcuate AgRP neurons. We found that 56.0 ± 3.2% (*n* = 6) of AgRP neurons express Fos when the mice were fasted overnight for 18 h (Fig 4A and 4B).

To address the contribution of GIRK1 and GIRK2 subunits, we generated conditional knockout mice by breeding *Agrp*-ires-Cre mice [21] and *Girk1*^flox/flox^ or *Girk2*^flox/flox^ mice [22,23]. We performed in situ hybridization (ISH) experiments (BaseScope) to confirm successful deletion of *Girk1* in the arcuate AgRP neurons of the *Agrp*-ires-Cre::*Girk1*^flox/flox^ (GIRK1^AgRP-KO^) mice compared to *Girk1*^flox/flox^ (GIRK1^WT^) mice (S7A–S7C Fig). We noted that *Girk2* expression by the arcuate AgRP neurons was not significantly different between GIRK1^WT^ and GIRK1^AgRP-KO^ mice (S7D Fig). We also examined *Girk1* expression in the hippocampus and confirmed that its expression is not affected by AgRP neuron-specific deletions (S8A and S8B Fig), which was further confirmed and quantified by qRT-PCR experiments (S8C Fig). Likewise, we confirmed a significant reduction of *Girk2*, but comparable expression of *Girk1*, in the arcuate AgRP neurons of the *Agrp*-ires-Cre::*Girk2*^flox/flox^ (GIRK2^AgRP-KO^) mice compared to *Girk2*^flox/flox^ (GIRK2^WT^) mice (S7E–S7H Fig) and observed comparable expression of *Girk2* in the hippocampus (S8D–S8F Fig). Together, these results indicate successful generation of conditional GIRK channel knockout models.

Subsequently, we measured Fos expression levels of tdTomato-expressing AgRP neurons in the ARH obtained from *Agrp*-ires-Cre::tdTomato::*Girk1*^flox/flox^ (*Agrp*^tdTomato^/*Girk1*^KO^) and *Agrp*-ires-Cre::tdTomato::*Girk2*^flox/flox^ (*Agrp*^tdTomato^/*Girk2*^KO^) mice that were fasted overnight for 18 h. We observed significantly increased levels of Fos expression by AgRP neurons from the *Agrp*^tdTomato^/*Girk2*^KO^ mice, where 71.7 ± 4.8% (*n* = 4) of AgRP neurons showed Fos immunoreactivity (*p* = 0.020, Fig 4A and 4B) compared to results obtained from *Agrp*^tdTomato^ mice. On the other hand, 64.6 ± 2.6% (*n* = 4) of AgRP neurons from the *Agrp*^tdTomato^/*Girk1*^KO^ mice expressed Fos, which was not significantly different from what was observed in the *Agrp*^tdTomato^ mice (*p* = 0.231, Fig 4A and 4B). These results are consistent with the depolarized RMP of NPY^G2KO^ neurons (Fig 3). Therefore, we suggest that at a population level GIRK2-containing GIRK channels, rather than GIRK1-containing GIRK channels, contribute to maintain AgRP neuronal activity.

### Deletion of GIRK2 subunits in AgRP neurons increases adiposity and body weight independently of food intake

**Fig 5:**
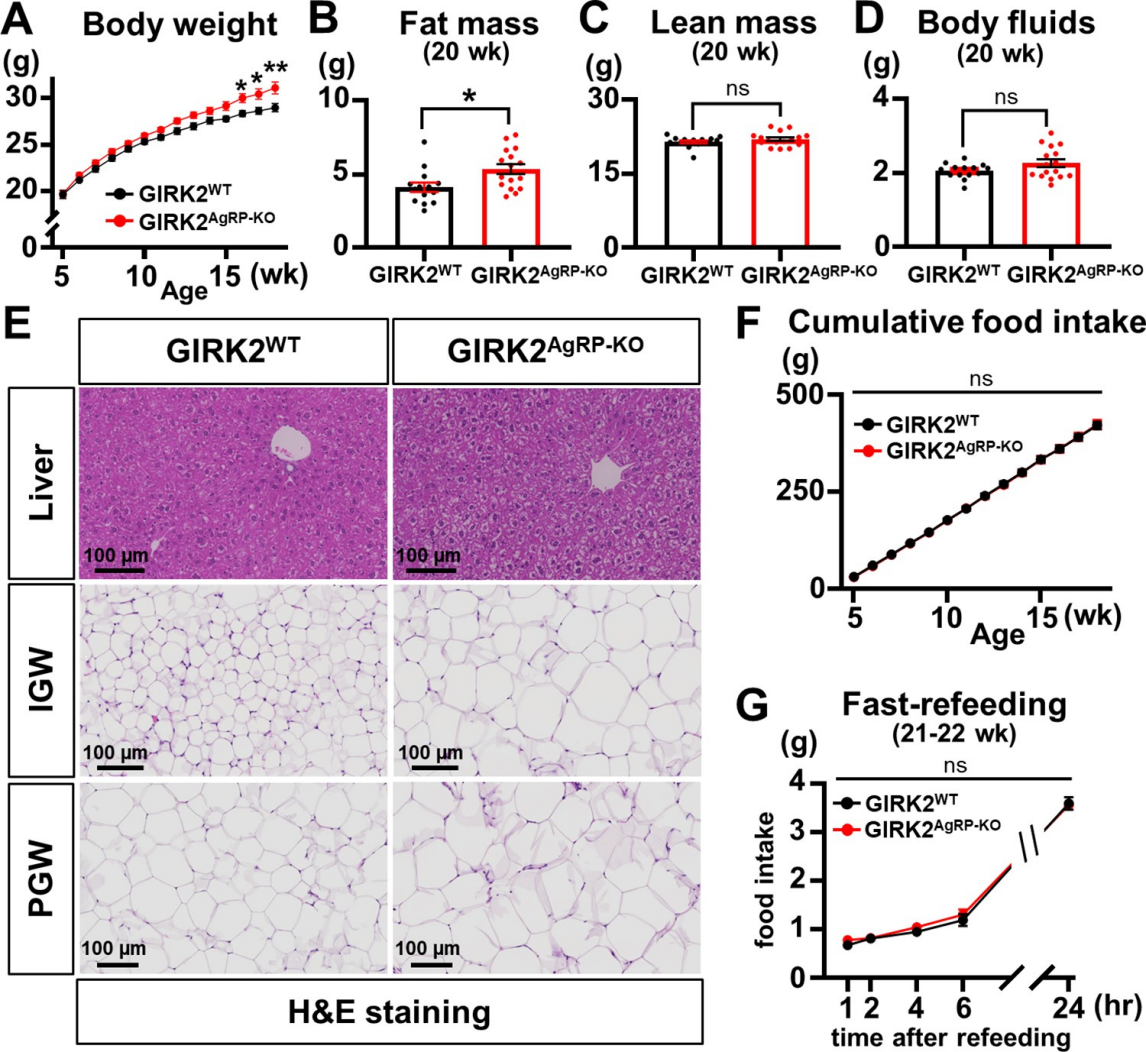
GIRK2^AgRP-KO^ mice show increased body weight and adiposity independently of food intake. (A) Body weights of GIRK2^WT^ (*n* = 14) and GIRK2^AgRP-KO^ (*n* = 16) mice on NCD. Two-way repeated measures ANOVA with Bonferroni correction, gene (df = 1, *F*_*1*, *28*_ *=* 5.251, and *p* = 0.030), time (df = 13, *F*_*13*, *364*_ = 416.6, and *p* < 0.0001), and interaction (df = 13, *F*_*13*, *364*_ = 3.372, and *p* < 0.0001). (B–D) Bar graphs and dots summarize fat mass (4.1 ± 0.3 g, *n* = 14, for GIRK2^WT^ and 5.4 ± 0.3 g, *n* = 16, for GIRK2^AgRP-KO^, df = 28, t = 2.627, *p* = 0.014) (B), lean mass (21.5 ± 0.3 g, *n* = 14, for GIRK2^WT^ and 22.0 ± 0.4 g, *n* = 16, for GIRK2^AgRP-KO^, df = 28, t = 1.113, *p* = 0.275) (C), and body fluids (2.1 ± 0.1 g, *n* = 14, for GIRK2^WT^ and 2.3 ± 0.1 g, *n* = 16, for GIRK2^AgRP-KO^, df = 28, *t* = 1.648, *p* = 0.111) (D) of GIRK2^WT^ (*n* = 14) and GIRK2^AgRP-KO^ (*n* = 16) mice by NMR spectrometer analyses. (E) Images demonstrate HE staining results of liver, IGW, and PGW obtained from GIRK2^WT^ and GIRK2^AgRP-KO^ mice. Scale bar = 100 μm. (F) Cumulative food intake of GIRK2^WT^ (*n* = 14) and GIRK2^AgRP-KO^ (*n* = 16) mice. Two-way repeated measures ANOVA with Bonferroni correction, gene (df = 1, *F*_*1*, *28*_ = 0.007, and *p* = 0.934), time (df = 13, *F*_*13*, *364*_ = 2196, and *p* < 0.0001), and interaction (df = 13, *F*_*13*, *364*_ = 0.0389, and *p* > 0.9999). (G) Food intake of GIRK2^WT^ (*n* = 8) and GIRK2^AgRP-KO^ (*n* = 9) mice in fast-refeeding experiments. Two-way repeated measures ANOVA with Bonferroni correction, gene (df = 1, *F*_*1*, *15*_ = 0.269, and *p* = 0.612), time (df = 4, *F*_*4*, *60*_ = 960.6, and *p* < 0.0001), and interaction (df = 4, *F*_*4*, *60*_ = 0.713, and *p* = 0.587). Data are presented as mean ± SEM. Two-way repeated measures ANOVA with Bonferroni correction (A, F, G) and unpaired *t* test (B–D) were used for statistical analyses. *p < 0.05, **p < 0.01, ns = not significant. The numerical data for Fig 5A–5D, 5F, and 5G can be found in S5 Data. GIRK, G protein-gated inwardly rectifying K^+^; HE, hematoxylin and eosin; IGW, inguinal white; NCD, normal chow diet; NMR, nuclear magnetic resonance; PGW, perigonadal white.

In order to delineate the metabolic function of GIRK2 subunits expressed by AgRP neurons, we measured body weight and food intake of GIRK2^AgRP-KO^ and GIRK2^WT^ mice once a week and found that GIRK2^AgRP-KO^ mice gained more body weight than GIRK2^WT^ mice on NCD (Fig 5A). The difference of body weight became more pronounced week by week to be statistically significant when the mice were 16 weeks old (Fig 5A). The nuclear magnetic resonance (NMR) analyses of body compositions, which was performed when the mice were 20 weeks old, demonstrated that the weight gain was due to increased fat mass (Fig 5B), while lean mass or body fluids were similar between genotypes (Fig 5C and 5D). Consistent with these findings, hematoxylin and eosin (HE) staining revealed infiltration of fat into the liver as well as increased size of adipocytes within the inguinal white (IGW) and perigonadal white (PGW) fat tissues of GIRK2^AgRP-KO^ mice (Fig 5E). We noted that differences in food consumption do not explain the increased adiposity, since cumulative food intake was not different between GIRK2^WT^ mice and GIRK2^AgRP-KO^ mice (Fig 5F). We also found that food intake was not influenced by GIRK2 deletion when the mice (21- to 22-week-old) were refed after overnight fasting (Fig 5G).

### GIRK2-containing GIRK channels expressed by AgRP neurons are required for normal sympathetic activity and BAT function

**Fig 6:**
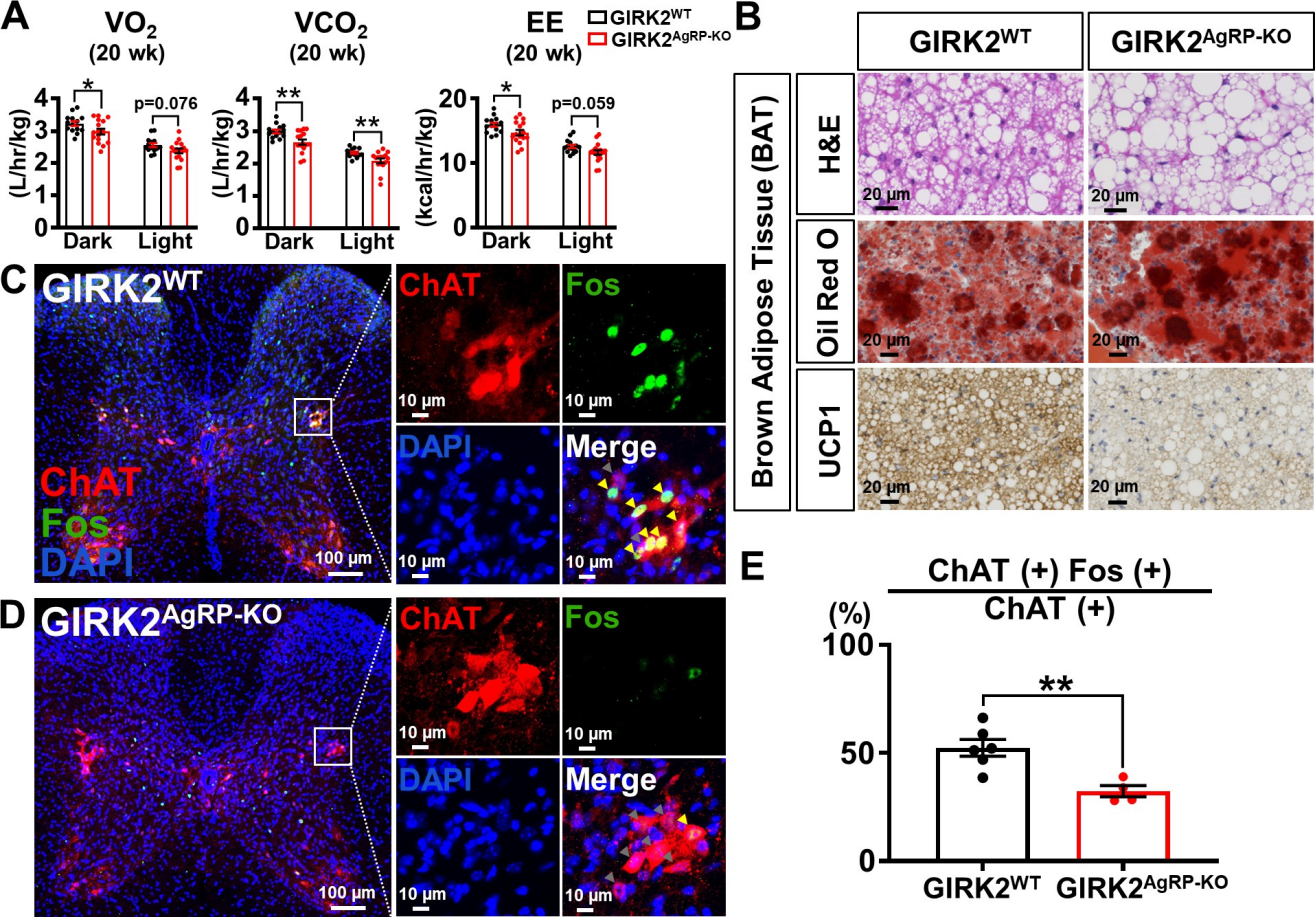
GIRK2^AgRP-KO^ mice show decreased EE associated with BAT dysfunction and decreased sympathetic activity. (A) Bar graphs and dots summarize oxygen consumption (VO_2_) (left, dark cycle: 3.23 ± 0.07 L/h/kg, *n* = 14, for GIRK2^WT^ and 2.98 ± 0.09 L/h/kg, *n* = 16, for GIRK2^AgRP-KO^, df = 28, t = 2.088, *p* = 0.046; light cycle: 2.57 ± 0.06 L/h/kg, *n* = 14, for GIRK2^WT^ and 2.38 ± 0.07 L/h/kg, *n* = 16, for GIRK2^AgRP-KO^, df = 28, t = 1.842, *p* = 0.076), carbon dioxide production (VCO_2_) (middle, dark cycle: 2.99 ± 0.06 L/h/kg, *n* = 14, for GIRK2^WT^ and 2.66 ± 0.08 L/h/kg, *n* = 16, for GIRK2^AgRP-KO^, df = 28, t = 3.198, *p* = 0.003; light cycle: 2.33 ± 0.04 L/h/kg, *n* = 14, for GIRK2^WT^ and 2.09 ± 0.07 L/h/kg, *n* = 16, for GIRK2^AgRP-KO^, df = 28, t = 2.847, *p* = 0.008), and EE (right, dark cycle: 15.9 ± 0.3 kcal/h/kg, *n* = 14, for GIRK2^WT^ and 14.7 ± 0.4 kcal/h/kg, *n* = 16, for GIRK2^AgRP-KO^, df = 28, t = 2.140, *p* = 0.041; light cycle: 12.6 ± 0.3 kcal/h/kg, *n* = 14, for GIRK2^WT^ and 11.7 ± 0.4 kcal/h/kg, *n* = 16, for GIRK2^AgRP-KO^, df = 28, t = 1.972, *p* = 0.059) of GIRK2^WT^ (*n* = 14) and GIRK2^AgRP-KO^ (*n* = 16) mice measured by indirect calorimetry. (B) Images demonstrate HE (upper), oil red O (middle) staining, and UCP1 (lower) immunostaining results of BAT obtained from GIRK2^WT^ (left) and GIRK2^AgRP-KO^ (right) mice. Scale bar = 20 μm. (C, D) Images on the left demonstrate IHC of ChAT (red), Fos (green), and DAPI (blue) in upper (T1-T6) thoracic spinal cords of GIRK2^WT^ (C) and GIRK2^AgRP-KO^ (D) mice at a lower magnification. Scale bar = 100 μm. Areas of IML in the rectangles are shown on the right at a higher magnification. In merged images, gray arrowheads indicate Fos (−) and ChAT (+) neurons, and yellow arrowheads indicate Fos (+) and ChAT (+) neurons. Scale bar = 10 μm. (E) Bar graphs and dots summarize proportion of Fos-expressing ChAT neurons in IML of GIRK2^WT^ (52.4 ± 3.9%, *n* = 6) and GIRK2^AgRP-KO^ (32.4 ± 2.6%, *n* = 4) mice (df = 8, t = 3.79, *p* = 0.005). A total of 48 spinal cord slices from each mouse (levels T1-T6) were included for analyses. Data are presented as mean ± SEM. Unpaired *t* test was used for statistical analyses. *p < 0.05, **p < 0.01. The numerical data for Fig 6A and 6E can be found in S6 Data. BAT, brown adipose tissue; ChAT, choline acetyltransferase; EE, energy expenditure; GIRK, G protein-gated inwardly rectifying K^+^; HE, hematoxylin and eosin; IHC, immunohistochemistry; IML, intermediolateral column.

Given no changes in food intake, we hypothesized that the body weight gain observed in GIRK2^AgRP-KO^ would be caused by decreased energy expenditure. To test this idea, we measured oxygen consumption (VO_2_) and carbon dioxide production (VCO_2_) with an indirect calorimetry from 20-week-old GIRK2^WT^ and GIRK2^AgRP-KO^ mice. We observed significantly decreased VO_2_ and VCO_2_ in GIRK2^AgRP-KO^ mice compared to GIRK2^WT^ mice (Fig 6A, left and middle). The calculated EE was also significantly decreased in the GIRK2^AgRP-KO^ mice (Fig 6A, right). During the indirect calorimetry measurements, we also measured ambulatory movements and rearing activities, but there was no difference between genotypes (S9A and S9B Fig). Both GIRK2^WT^ mice and GIRK2^AgRP-KO^ mice (21- to 22-weeks-old) moved similar distance when they were allowed to move freely in chambers designed for an open field test (OFT) (S9C and S9D Fig). AgRP neurons were shown to regulate anxiety level [24], but our OFT results demonstrated similar levels of anxiety regardless of genotypes, based on their comparable preference to the center zone and the outer zone in the chamber (S9E–S9G Fig). Thus, the decreases in EE observed in GIRK2^AgRP-KO^ mice are likely due to reduced basal metabolic rate.

Decreased BAT thermogenesis is often a major cause of reduced basal metabolic rate and energy expenditure [25]. Indeed, we noted increased adiposity and triacylglycerol level in the BAT from the GIRK2^AgRP-KO^ mice by HE and oil red O staining (Fig 6B, top and middle). In addition, uncoupling protein-1 (UCP-1) immunoreactivity was markedly decreased in the BAT of GIRK2^AgRP-KO^ mice (Fig 6B, bottom). Since BAT thermogenesis is regulated by sympathetic tone [26] and NPY/AgRP neurons are known to decrease sympathetic activity [5,27,28], we predicted that increased activity of NPY/AgRP neurons would result in decreased sympathetic activity of GIRK2^AgRP-KO^ mice. To test this idea, we performed IHC experiments and measured Fos levels in the cholinergic sympathetic preganglionic neurons of the intermediolateral column (IML) of T1 to T6 spinal cords. We found in GIRK2^AgRP-KO^ mice a significantly lower percentage (32.4 ± 2.6%, *n* = 4, *p* = 0.005) of choline acetyltransferase (ChAT)-positive IML neurons expressing Fos compared to observations in the GIRK2^WT^ mice (52.4 ± 3.9%, *n* = 6) (Fig 6C–6E) at 8 to 12 weeks of age. Together, these results suggest that decreased sympathetic activity and BAT thermogenesis lead to decreased energy expenditure and body weight gain in GIRK2^AgRP-KO^ mice.

### GIRK2 expressed by AgRP neurons is necessary for prompt adaptation to a cold temperature

Our results suggested that GIRK2 subunits expressed by AgRP neurons contribute to maintain body weight by promoting EE in non-stress conditions. To explore if GIRK2 subunits also have a role in stress conditions, we intraperitoneally (i.p.) injected 10-week-old GIRK2^WT^ and GIRK2^AgRP-KO^ mice with ghrelin (0.4 mg/kg) and measured food intake for 4 h after injections. We expected that ghrelin produces hunger-induced stress, but found that ghrelin-induced increase of food intake was similar between GIRK2^WT^ and GIRK2^AgRP-KO^ mice (S10 Fig). GIRK2^WT^ and GIRK2^AgRP-KO^ mice used for this experiment weighed 27.7 ± 1.0 g (*n* = 4) and 28.1 ± 1.2 g (*n* = 4), respectively (*p* > 0.5 by unpaired *t* test).

**Fig 7:**
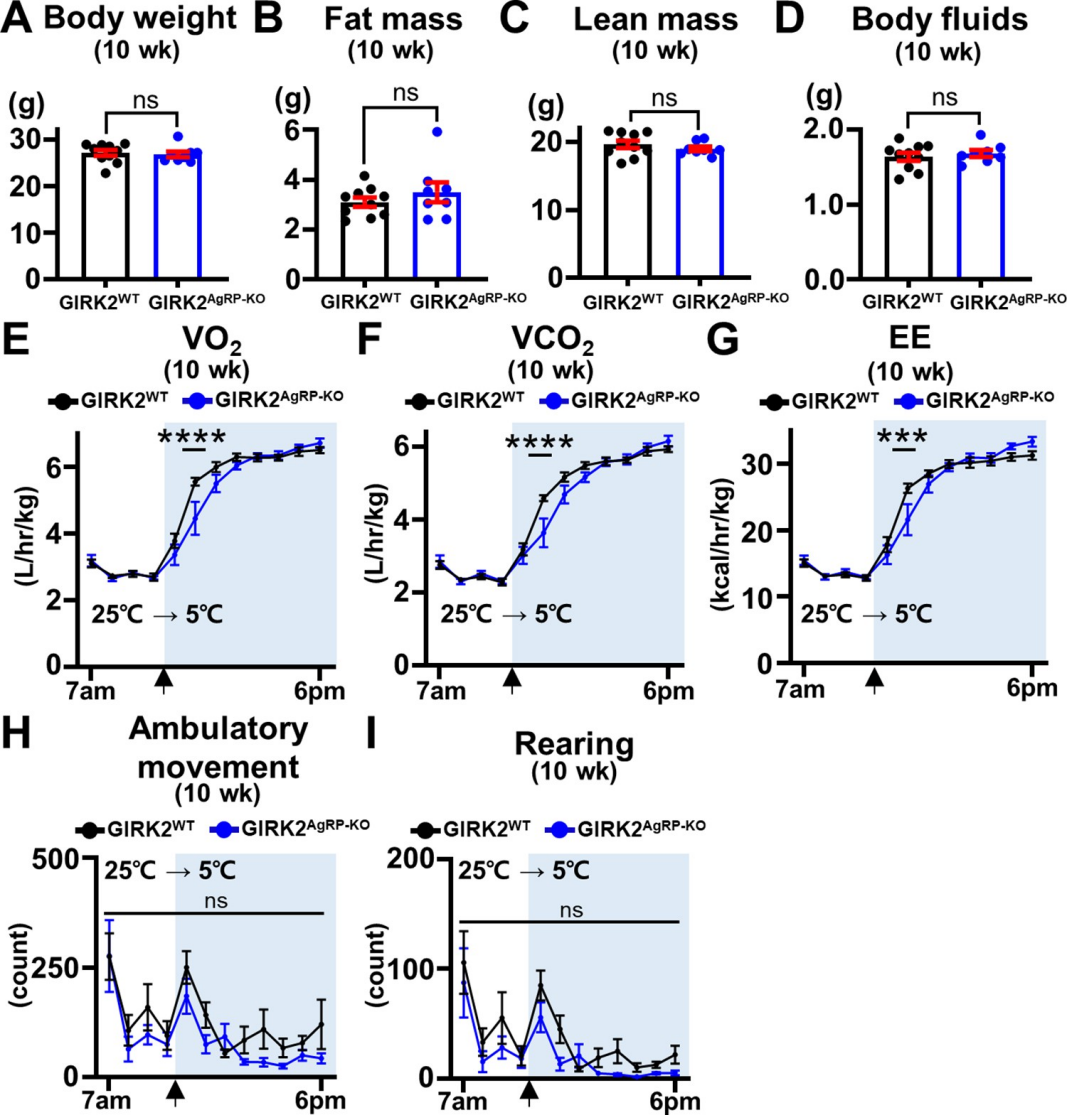
GIRK2^AgRP-KO^ mice show compromised adaptation to cold exposure. (A–D) Bar graphs and dots summarize body weight (27.1 ± 0.6 g, *n* = 10, for GIRK2^WT^ and 26.8 ± 0.6 g, *n* = 8, for GIRK2^AgRP-KO^, df = 16, t = 0.380, *p* = 0.706) (A), fat mass (3.1 ± 0.2 g, *n* = 10, for GIRK2^WT^ and 3.5 ± 0.4 g, *n* = 8, for GIRK2^AgRP-KO^, df = 16, *t* = 0.967, *p* = 0.348) (B), lean mass (19.7 ± 0.6 g, *n* = 10, for GIRK2^WT^ and 19.0 ± 0.3 g, *n* = 8, for GIRK2^AgRP-KO^, df = 16, t = 0.936, *p* = 0.364) (C), and body fluids (1.6 ± 0.1 g, *n* = 10, for GIRK2^WT^ and 1.7 ± 0.0 g, *n* = 8, for GIRK2^AgRP-KO^, df = 16, t = 0.587, *p* = 0.566) (D) of 10-week-old male GIRK2^WT^ mice (*n* = 10) and GIRK2^AgRP-KO^ mice (*n* = 8) on NCD before cold exposure. (E–I) Graphs summarize oxygen consumption (VO_2_) (E), carbon dioxide production (VCO_2_) (F), EE (G), ambulatory movement (H), and rearing activity (I) of 10-week-old male GIRK2^WT^ mice (*n* = 10) and GIRK2^AgRP-KO^ (*n* = 8) in response to cold exposure (from 25°C to 5°C). Mice were acclimated for 2 days before experiments. Unpaired *t* test was used for statistical analyses (A–D), and two-way repeated measures ANOVA with Bonferroni correction was used for statistical analysis (E–I). VO_2_ (E): gene (df = 1, *F*_*1*,*16*_ = 1.670, *p* = 0.215), time (df = 11, *F*_*11*, *176*_ = 267.1, *p* < 0.0001), and interaction (df = 11, *F*_*11*, *176*_ = 3.237, *p* = 0.0005). VCO_2_ (F): gene (df = 1, *F*_*1*,*16*_ = 1.565, *p* = 0.229), time (df = 11, *F*_*11*, *176*_ = 284.1, *p* < 0.0001), and interaction (df = 11, *F*_*11*, *176*_ = 3.226, *p* = 0.0005). EE (G): gene (df = 1, *F*_*1*,*16*_ = 0.110, *p* = 0.744), time (df = 11, *F*_*11*, *176*_ = 257.1, *p* < 0.0001), and interaction (df = 11, *F*_*11*, *176*_ = 3.075, *p* = 0.0008). Ambulatory movement (H): gene (df = 1, *F*_*1*,*16*_ = 1.38, *p* = 0.257), time (df = 11, *F*_*11*, *176*_ = 12.87, *p* < 0.0001), and interaction (df = 11, *F*_*11*, *176*_ = 0.787, *p* = 0.653). Rearing activity (I): gene (df = 1, *F*_*1*,*16*_ = 2.391, *p* = 0.142), time (df = 11, *F*_*11*, *176*_ = 11.51, *p* < 0.0001), and interaction (df = 11, *F*_*11*, *176*_ = 0.569, *p* = 0.852). ***p < 0.001, ****p < 0.0001, ns = not significant. The numerical data for Fig 7A–7I can be found in S7 Data. EE, energy expenditure; GIRK, G protein-gated inwardly rectifying K^+^; NCD, normal chow diet.

In a different set of experiments, we exposed 10-week-old GIRK2^WT^ and GIRK2^AgRP-KO^ mice to a cold environment (5°C) to challenge the mice with cold stress. There was no significant difference in body weight or body composition between the genotypes (Fig 7A–7D). When the temperature dropped from 25°C to 5°C, GIRK2^WT^ mice showed a prompt increase of VO_2_ and VCO_2_, which reached a new steady state after approximately 4 h (Fig 7E and 7F). GIRK2^AgRP-KO^ mice also showed increase of VO_2_ and VCO_2_ in response to the cold exposure, but there was a significant delay in the rising phase of VO_2_ and VCO_2_ (Fig 7E and 7F). The calculated EE were also significantly different in the rising phase between the genotypes (Fig 7G). We noted no significant differences in ambulatory movement or rearing activity of GIRK2^WT^ and GIRK2^AgRP-KO^ mice (Fig 7H and 7I), suggesting that the increases of VO_2_ and VCO_2_ are likely from increased BAT thermogenesis. Taken together, we propose that GIRK2 expressed by AgRP neurons is dispensable for ghrelin-induced feeding, but is necessary for prompt adaptation to a cold environment.

## Discussion

In this study, we found evidence that GIRK2 subunits are key to regulating the long-term baseline activity of arcuate NPY/AgRP neurons. In agreement, GIRK2 ablation in arcuate NPY/AgRP neurons resulted in increased adiposity and body weight. This phenotype was associated with decreased sympathetic activity and reduced energy expenditure, but not changes in food intake. We also demonstrated that GIRK2 ablation in arcuate NPY/AgRP neurons delays the initial phase of cold-induced thermogenesis. Together, our findings identified GIRK2 as a regulator of arcuate NPY/AgRP neuron activity that maintains sympathetic activity and burns fat, which should help to maintain homeostasis in physiological (normal caloric or non-stressed) and some stressed conditions.

### In vivo metabolic effects of AgRP neuron activity

Previous studies demonstrated that the optogenetic or chemogenetic activation of AgRP neurons results in increased food intake and/or decreased energy expenditure [6,7,29], which occurred within hours. The activation of AgRP neurons using the “capsaicin-Trpv1” system also resulted in rapid decreases of energy expenditure and thermogenesis [30]. On the other hand, chemogenetic inhibition of AgRP neurons for 14 days led to decreased body weight, decreased food intake, and fat burning [31]. It was also shown that chemogenetic inhibition of AgRP neurons reverses diabetes-induced hyperphagia and hyperglycemia within a few hours [32]. Therefore, available data suggested that modulation of AgRP neuronal activity can significantly affect food intake and energy expenditure in time frames of hours to days.

Since acute activation of AgRP neurons resulted in rapid metabolic effects regardless of activating methods, we may expect that long-term activation of AgRP neurons would also produce similar phenotypes. A recent study overexpressed bacterial sodium channel (NachBac) or Kir2.1 channel selectively in AgRP neurons to achieve long-term activation and inhibition of neuronal activity, respectively [33]. The authors reported that NachBac overexpression resulted in massive obesity accompanied by increased food intake but no changes of energy expenditure, but that Kir2.1 overexpression did not produce any phenotype. In this study, we genetically deleted GIRK2 subunits selectively in the AgRP neurons, which presumably increased AgRP neuronal activity for longer periods (approximately 5 months). While we noted significantly increased body weight in GIRK2^AgRP-KO^ mice, cumulative food intake was not different between genotypes. We also noted that fasting- and ghrelin-induced feeding were not different between genotypes. This finding was quite surprising given the prominent role of AgRP neurons in the regulation of food intake. Instead, O_2_ consumption and CO_2_ production were significantly decreased in GIRK2^AgRP-KO^ mice, which were associated with decreased activity of sympathetic preganglionic neurons and decreased UCP-1 expression by BAT. GIRK2^AgRP-KO^ mice also showed a significant delay in cold-induced increase of energy expenditure compared to GIRK2^WT^ mice (Fig 7), which further suggested that thermogenic response is compromised in GIRK2^AgRP-KO^ mice.

It is not clear why persistently increased activity of AgRP neurons decreased energy expenditure but did not regulate food intake in our study. One hypothesis is that GIRK2-expressing AgRP neurons preferentially regulate energy expenditure, like leptin receptor-expressing POMC neurons [34]. In this scenario, food intake and energy expenditure are regulated by distinct subpopulations of AgRP neurons, as previously suggested for POMC neurons [35]. An alternative possibility is that GIRK2-expressing AgRP neurons also regulate food intake, but this effect is discernable only in short-term time frames. In other words, AgRP neurons can regulate both food intake and energy expenditure, but a compensatory anorexia (due to decreased energy expenditure) may develop over time to mask increased food intake. In either case, the inhibition of energy expenditure by GIRK2-ablated AgRP neurons looks large enough. It is also important to note that we deleted GIRK2 subunits before birth. As GIRK channels can be assembled in 5 different compositions [36,37], other GIRK channel subunits may take the role of GIRK2 subunits in AgRP neurons in GIRK2^AgRP-KO^ mice. Therefore, we may need a strategy to delete GIRK2 subunits postnatally to delineate the actual function of GIRK2 subunits expressed by AgRP neurons. Indeed, a previous study suggested that a compensatory mechanism may develop before birth to overcome the severe anorexia observed in AgRP-ablated mice [38]. We suggest that future studies are directed to delineate the metabolic functions designated to individual AgRP neurons, which will help to understand variable phenotypes obtained from AgRP neuron-specific conditional knockout mouse models.

### Metabolic function of K^+^ channels expressed by NPY/AgRP neurons

A few previous studies reported the role of K^+^ channels expressed by the AgRP neurons in the regulation of energy balance. For example, a study demonstrated that down-regulation of the small conductance Ca^2+^-activated K^+^ (SK) channels contribute to fasting-induced activation of AgRP neurons [39]. It was noted that SK3 channel deletion resulted in increased firing rate of AgRP neurons without changes in RMP, which makes sense given the role of SK channel in the regulation of afterhyperpolarization. AgRP neuron-specific deletions of SK3 channels resulted in a transient obesity in NCD-fed mice, but profoundly exacerbated HFD-induced obesity. The increased susceptibility to HFD was associated with increased food intake and decreased EE, while the locomotive activity remained unchanged. More recently, it was shown that CRISPR knockdown of *Kcnq3*, an M channel subunit, in NPY/AgRP neurons did not affect RMP but increased input resistance and decreased rheobase current, which suggested increased response to external stimuli [40]. However, it was noted that in NCD conditions KCNQ3 deficiency resulted in no changes of food intake and body weight, while locomotive activity was decreased. Similar results were obtained in HFD-fed mice except that there was an increase of abdominal fat mass. Together, it is suggested that M channels expressed by NPY/AgRP neurons are largely dispensable for the control of energy balance whether the mice are given NCD or HFD.

In this study, we found a predominant role for GIRK channels to maintain RMP of NPY/AgRP neurons (Fig 1). Notably, knockout of GIRK2 resulted in a depolarized RMP (approximately 3.5 mV), amplitude of which was comparable to the depolarizing effects of the GIRK channel blocker (approximately 4 mV). We also noted a few cells depolarized by M channel blockers, but a majority of NPY/AgRP neurons did not respond (S2 Fig), which is consistent with the limited in vivo function of KCNQ3 expressed by NPY/AgRP neurons [40]. While SK3 and KCNQ3 expressed by the AgRP neurons were largely dispensable in NCD-fed mice [39,40], GIRK2 ablation in AgRP neurons led to significantly increased body weight in NCD-fed mice (Fig 5). Together, it appears that GIRK2-containing GIRK channels have a unique role in the physiological NCD-fed conditions to regulate NPY/AgRP neuronal excitability and energy balance.

### Functional GIRK channel subunit composition in NPY/AgRP neurons

GIRK1/GIRK2 heterotetramers are the prototypes of neuronal GIRK channels, and loss of either GIRK1 or GIRK2 is usually sufficient to eliminate most or all of GIRK channel activity in central neurons [36,37]. A notable exception is the GIRK channel of midbrain dopaminergic neurons. They lack GIRK1 subunits, and GIRK2 homotetramers and/or GIRK2/GIRK3 heterotetramers are believed to be the subunit composition of GIRK channels in these neurons [36,37]. In this study, we found that GIRK2 is the major GIRK channel subunit expressed by arcuate AgRP neurons (Figs 2 and S3). Analyses of our FISH data suggest that GIRK2 homotetramer (and GIRK2/GIRK3 heterotetramer) may constitute a majority of functional GIRK channels in AgRP neurons (S3 Fig). Therefore, GIRK channel subunit composition of AgRP neurons appear to be similar to that of midbrain dopaminergic neurons.

We unexpectedly found that ablation of GIRK2 did not affect GABA_B_-activated K^+^ currents in NPY/AgRP neurons (S4 Fig). On the other hand, GIRK2-containing GIRK channels contributed to long-term control of NPY/AgRP neuronal excitability and in vivo energy balance (Figs 4–6). Together, these data may suggest that GIRK2-containing GIRK channels are open at rest to regulate in vivo metabolic function but are not functionally coupled to GABA_B_ receptor stimulation. In this case, GIRK1/GIRK3 and/or GIRK1/GIRK4 heterotetramers may be responsible for GABA_B_-activated K^+^ currents. However, we also need to consider the possibility that the loss of GIRK2 subunits and the contribution to GABA_B_-activated K^+^ currents was replaced by other GIRK subunits through a compensatory mechanism in our model. This is especially so given that almost all NPY/AgRP neurons generate outward currents by baclofen (S4 Fig) but *Girk1*/*Girk3* (7.7 ± 0.7%, *n* = 3) and *Girk1*/*Girk4* (8.1 ± 0.2%, *n* = 3) co-expression levels in NPY/AgRP neurons are quite low (S3B and S3C Fig). It remains to be tested whether GIRK2-containing GIRK channels are coupled to other G_i/o_ protein-coupled receptors. Overall, more investigations are necessary to delineate the subunit composition of functional GIRK channels of NPY/AgRP neurons.

## Materials and methods

### Ethics statement

All experiments were performed in accordance with the guidelines established by the Korea Advanced Institute of Science and Technology (KAIST) Institutional Animal Care and Use Committee (IACUC) (Protocol No. KA2021-126). KAIST IACUC follows the standard operating guidelines for IACUC established by the Animal and Plant Quarantine Agency and the Ministry of Food and Drug Safety of South Korea.

### Mice

All mice used for breeding and experiments in this study were housed in a light-dark (12 h on/off; lights on at 7:00 AM) and temperature-controlled environment with food and water available ad libitum in the KAIST facilities. *Npy*-hrGFP mice were obtained from the Jackson laboratory (#006417). For some patch clamp experiments GIRK2 KO mice [41], used with the permission from Dr. Markus Stoffel (ETH Zurich), were crossed with *Npy*-hrGFP mice. *Agrp*-ires-Cre mice (Jackson laboratory, #012899) were crossed with tdTomato reporter mice (Jackson laboratory, #007914), GIRK1^flox/flox^ mice [22] or GIRK2^flox/flox^ mice [23] for FISH, ISH, IHC, and in vivo metabolic experiments. Mice were fed standard NCD (Teklad global 18% protein 2018S, ENVIGO).

### Electrophysiology

Five- to 13-week-old male *Npy*-hrGFP mice were used for all patch clamp experiments in order to identify NPY-expressing neurons in the ARH. *Npy*-hrGFP mice were fasted for 18 h before being killed for experiments. Whole-cell patch clamp recordings from hrGFP-expressing neurons were maintained in acute hypothalamic slice preparations as previously described [42]. In brief, mice were deeply anesthetized with isoflurane inhalation and transcardially perfused with a modified ice-cold artificial CSF (ACSF) (described below), in which an equiosmolar amount of sucrose was substituted for NaCl. The mice were then decapitated, and the entire brain was removed from the skull and immediately submerged in ice-cold, carbogen-saturated (95% O_2_ and 5% CO_2_) ACSF (123 mM NaCl, 26 mM NaHCO_3_, 2.8 mM KCl, 1.25 mM NaH_2_PO_4_, 1.2 mM MgSO_4_, 2.5 mM CaCl_2_, and 10 mM glucose). A brain block containing the hypothalamus was made. Coronal sections (250 μm) were cut with a Leica VT1200S vibrating microtome and then incubated in oxygenated ACSF at 34°C for at least 1 h before recording. Brain slices were transferred to the recording chamber and allowed to equilibrate for 10 to 20 min before recording. The slices were bathed in oxygenated ACSF (32°C to 34°C) at a flow rate of approximately 2 ml/min. The pipette solution was modified to include an intracellular dye (Alexa Fluor 594) for whole-cell patch clamp recording: 120 mM K-gluconate, 10 mM KCl, 10 mM HEPES, 1 mM CaCl_2_, 1 mM MgCl_2_, 5 mM EGTA, 2 mM Mg-ATP, and 0.03 mM Alexa Fluor 594 hydrazide dye (pH 7.3). Epifluorescence was briefly used to target fluorescent cells, at which time the light source was switched to infrared differential interference contrast imaging to obtain the whole-cell recording (Nikon Eclipse FN1 equipped with a fixed stage and an optiMOS scientific CMOS camera). Recording electrodes had resistances of 3 to 5 MΩ when filled with the K-gluconate internal solution.

In current clamp experiments, input resistance was assessed by measuring amplitudes of voltage deflections in response to hyperpolarizing rectangular current step pulses (500 ms, −25 pA to 0 pA by 5 pA increments or −50 pA to 0 pA by 10 pA increments) which was applied at a stable membrane potential before and after drug application. AP threshold was determined from averaged AP traces of firing neurons. The voltage at the last minimum of dV/dt preceding the spike (within 2 ms preceding 10 V/s) was estimated to be AP threshold, as described previously [43]. A drug effect was required to be associated temporally with drug application, and the responses had to be stable within a few minutes. We determined membrane potential before (control) and during drug applications (drug) by averaging membrane potential for 10 s in each condition. A neuron was considered to be depolarized or hyperpolarized if a change in membrane potential was larger than 2 mV in amplitude. Membrane potentials were not compensated for liquid junction potentials (−8 mV).

For voltage clamp experiments, we used the same K-gluconate pipette solutions described above and added 0.5 μM tetrodotoxin (TTX) and synaptic blockers (50 μM picrotoxin and 1 mM kynurenic acid) to bath solutions. We held the membrane potential at −40 mV and locally applied baclofen using micropipettes attached to the Picospritzer III microinjection dispense system (Parker Hannifin). We placed micropipettes 10 to 20 μm away from soma and ejected small volume (15 to 20 pL) of ACSF containing baclofen and the blocker cocktail with a pressure of 16 to 18 psi for 15 s. Baclofen-activated currents (*I*_Bac_) were normalized by cell capacitance. Voltage ramp pulses (from −120 mV to −10 mV, 100 mV/s) were applied before and after baclofen applications from a holding potential of −40 mV to obtain I-V relationships of *I*_Bac_.

### In situ hybridization

In situ hybridization experiments were performed using RNAscope or BaseScope assays available from the Advanced Cell Diagnostics Inc. (Hayward, California, United States of America). Briefly, 8- to 12-week-old male mice were deeply anesthetized with isoflurane and transcardially perfused with a DEPC-treated PBS and subsequently with a 4% paraformaldehyde (163–20145, FUJIFILM Wako). Brains were removed from the skull and submerged in cold 4% paraformaldehyde solutions for 24 h (post-fixation). Brains were then transferred to a series of sucrose solutions of gradients (4 h in 10% sucrose, 12 h in 20% sucrose, and 24 h in 30% sucrose) at 4°C. Brain slices with 10 μm thickness were obtained using a cryostat (Leica) and were stored in a cryo-protectant. We collected coronal sections that contain arcuate nucleus based on the shape of third ventricle, median eminence, and hippocampus referring to the Allen Brain Atlas. Brain slices were transferred to a well plate (20 wells, 4 rows × 5 columns) immediately after sectioning one by one in a rostrocaudal order. We used rows from left to right and columns from up to down. After collecting the first 20 slices, we repeated the same procedure 5 to 7 times so that one well contains 5 to 7 brain slices each of which is spaced by 200 μm. We used 1 column for 1 set of experiments. Brain slices were mounted on glass slides (Superfrost Plus Microscope Slides, Thermo Fisher) for the RNAscope or the BaseScope assays. All reagents for these assays were purchased from Advanced Cell Diagnostics.

*AgRP* probe (target region 11–764, accession # NM_001271806.1), *Girk1* probe (target region 658–1679, accession # NM_008426.2), *Girk2* probe (target region 282–1456, accession # NM_001025584.2), *Girk3* probe (target region 84–1276, accession # NM_008429.2), and *Girk4* probe (target region 523–1781, accession # NM_010605.4) were used for the triple RNAscope Multiplex Fluorescent Assay. *AgRP* probe (target region 61–214, 2 pairs, accession # NM_007427.3), *Girk1* probe (target region 1351–1479, accession # NM_001355118.1), and *Girk2* probe (target region 560–678, accession # NM_001025585.2) were used for the BaseScope Duplex Assay. *Girk1* and *Girk2* probes were custom-designed based on the targeted sequences of the *Girk1*^flox/flox^ and the *Girk2*^flox/flox^ mice [22,23]. Images of the RNAscope and BaseScope assays were obtained with a confocal microscope (LSM 780, Carl Zeiss) and a slide scanner (Axio Scan. Z1, Carl Zeiss), respectively, and were analyzed with ZEN lite (ZEN Microscopy software) and the Image J software. We lined up all stained images from individual mice, based on the reference brain atlas (Allen Brain Atlas), and selected 12 or 16 brain slices (from each mouse) that are available from all mice to be included for analyses.

### RNA extraction and qRT-PCR

Brain blocks were prepared and submerged in ice-cold ACSF. Coronal brain slices (500 μm-1 mm thickness) were obtained using Leica VT1200S vibrating microtome. The brain slices were transferred to DEPC-based PBS on ice, and hippocampal regions containing dentate gyrus and CA1 were punched out using a blunt-end 16 gauge needle (#28110, STEMCELL Technologies) under a stereomicroscope. Hippocampal tissues were transferred to E-tubes on dry ice and preserved at −80°C. RNA was extracted from the hippocampal tissues by using RNeasy Lipid Tissue Mini Kit (74804, Qiagen).

qRT-PCR operation and analysis were performed as reported previously [44]. Briefly, 2 μg of RNA was added to master mix (SuperiorScript III RT Master Mix, RT300S, Enzynomics) and autoclaved de-ionized water to be 20 μl volume of mixture in total. We used a polymerase chain reaction (PCR) protocol, 25°C (10 min)– 42°C (1 h)– 85°C (5 min), for the synthesis of first-strand complimentary DNA (cDNA) synthesis. Product of PCR was diluted with autoclaved de-ionized water to be 100 μl. Approximately 1 μl of cDNA was mixed with SYBR Green Realtime PCR Master Mix (QPK-201, TOYOBO), target primers, and autoclaved de-ionized water to be 20 μl volume. Cycle threshold (C_t_) method was used for quantitative analysis of target gene mRNA. Relative expressional levels of target genes were determined by comparing the level to that of 2 housekeeping genes: GAPDH and 18S genes. Primers targeting GIRK channel subunits and protocols for amplification were prepared as described previously [45]. The primers used are as follows: *Girk1(forward)* 5′-GAGGGACGGAAAACTCACTCT-3′; *Girk1(reverse)* 5′-TCAGGTGTCTGCCGAGATT-3′; *Girk2(forward)* 5′-CGTGGAGTGAATTATTGAATCT-3′; *Girk2(reverse)* 5′-GTCATTTCTTCTTTGTGCTTTT-3′. We used an amplification protocol of 95°C (5 min, 1 cycle)– 45 cycles of 95°C (10 s)– 60°C (30 s)– 72°C (10 s).

### Immunohistochemistry

Fos activity in the hypothalamus and the spinal cord was detected using IHC experiments. Briefly, 8- to 12-week-old male mice were deeply anesthetized with isoflurane and transcardially perfused with a PBS and subsequently with a 4% paraformaldehyde (163–20145, FUJIFILM Wako).

Brains were obtained from the *Agrp*^*tdTomato*^, *Agrp*^*tdTomato*^*/Girk1*^*KO*^, and *Agrp*^*tdTomato*^*/Girk2*^*KO*^ mice and submerged in cold 4% paraformaldehyde for 24 h (post-fixation). Brains were then transferred to a series of sucrose solutions (4 h in 10% sucrose, 12 h in 20% sucrose, and 24 h in 30% sucrose) at 4°C. Brain slices with 15 μm thickness were obtained using a cryostat (Leica) and were prepared for incubation with primary antibodies. Collection of brain slices were performed as described in the methods for in situ hybridization experiments, except that brain slices in 1 well are spaced by 300 μm. Brain slices were mounted on glass slides (Superfrost Plus Microscope Slides, Thermo Fisher) and were treated with PBS containing 10% BSA and 0.3% Triton X-100 (blocking solution). The slices were subsequently incubated with anti-Fos (1:2,000, ab190289, Abcam) antibodies overnight at 4°C. Brain slices were then washed 3 times with PBS (10 min each) and were incubated with Alexa Fluor 647 donkey anti-rabbit secondary antibodies (Thermo Fisher Scientific) for 1 h at room temperature (RT) for immunofluorescence detection. The slices were incubated with DAPI for 10 min, washed 3 times with PBS (10 min each), and cover-slipped using fluorescence mounting medium (DAKO). Images were obtained with a confocal microscope (LSM 780, Carl Zeiss) and were analyzed with ZEN lite (ZEN Microscopy software) and Image J software.

Thoracic spinal cords obtained from GIRK2^WT^ and GIRK2^AgRP-KO^ mice were submerged in cold 4% paraformaldehyde for 12 h (post-fixation). Spinal cords were then transferred to 30% sucrose solutions for 24 h at 4°C. Spinal cord slices with 40 μm thickness were obtained using a cryostat (Leica). We collected coronal sections beginning from the cervical enlargements to preserve T1 level. Spinal cord slices were transferred to 24-well plates, placing 5 consecutive slices in 1 well. Since thoracic spinal cord (T1-L1) is about 18.2 mm in length [46], we needed 4 plates for 1 mouse spinal cord. We randomly selected 1 slice from 1 well, which allows average spacing of 200 μm between slices. Selected slices were washed 3 times with PBS (10 min each) to be prepared for incubation with primary antibodies. The slices were mounted on adhesive microscope slides (TruBond 380, Electron Microscopy Science) and were washed 3 times with PBS (10 min each). The slices then underwent heat-induced epitope retrieval at 60°C for 30 min and were treated with PBS containing 5% normal donkey serum (NDS) and 0.3% Triton X-100 (blocking solution). Subsequently, the slices were treated with anti-ChAT (1:100, AB144P, Sigma) antibody diluted with the blocking solution overnight at 4°C, which was followed by 1 h treatment with Alexa Fluor 488 donkey anti-goat secondary antibodies (Thermo Fisher Scientific) at RT. After that, the slices were treated with the blocking solution for 1 h, and then with anti-Fos (1:500, ab190289, Abcam) antibodies overnight at 4°C. The slices were then treated with Alexa Fluor 647 donkey anti-rabbit secondary antibodies (Thermo Fisher Scientific) for 1 h at RT. The slices were incubated with DAPI for 10 min and were washed 3 times with PBS (10 min each). Then, the slices were cover-slipped using fluorescence mounting medium (DAKO). Images were obtained with a confocal microscope LSM 780 (Carl Zeiss) and were analyzed with ZEN lite (ZEN Microscopy software) and Image J software. We examined the images to look for red fluorescence-expressing neurons in the IML since the first slice with positive fluorescence is T1. We proceeded starting from that slice to determine the level of each spinal cord section. We selected 48 spinal cord slices (approximately 9.6 mm in length, T1-T6, from each mouse) to be included for analyses.

UCP1 in the BAT was detected using IHC experiments. Briefly, 22- to 23-week-old male mice were deeply anesthetized with isoflurane. BAT was obtained from GIRK2^WT^ and GIRK2^AgRP-KO^ mice and was immediately submerged into formalin (HT501128, Sigma) for at least 24 h. Sections of BAT were treated with recombinant anti-UCP1 antibody (1:1,000, ab234430, Abcam), which was followed by incubation with horseradish peroxidase (HRP) secondary antibodies (Envision kit HRP, DAKO). Images were obtained with a slide scanner (Axio Scan. Z1, Carl Zeiss).

### Body weight and food intake

Body weight and food intake were measured from male mice as indicated in results. Each conditional knockout mouse (GIRK2^AgRP-KO^) had its littermate control mouse (GIRK2^WT^), and 21- to 22-week-old male mice were fasted for 18 h for fast-refeeding experiments. Refeeding started at 10 AM. For some experiments, 10-week-old male mice were i.p. injected with saline or ghrelin (0.4 mg/kg) in fed state (10 AM).

### Energy expenditure, physical activity, and body composition

Energy expenditure and physical activity were measured from 20-week-old male mice by an indirect calorimetric chamber (CLAMS 12; Columbus Instruments, Columbus, Ohio, USA). After acclimation for 2 days, O_2_ consumption (VO_2_) and CO_2_ production (VCO_2_) were measured for 2 days to determine the energy expenditure. Simultaneously, physical activity was determined using a multidimensional infrared light beam system with beams installed on bottom and top levels of cage. Ambulatory movement was defined as breaks of any 2 different light beams at bottom level of cage, while rearing was recorded once the mouse broke any light beam at the top level. Body composition was measured by Time Domain (TD) NMR spectrometer (Minispec LF50, Bruker biospin, Rheinstetten, Germany). For some experiments, we exposed mice (10 weeks old) to cold environment by changing the temperature to 5°C from 25°C, which occurred at 10:30 AM. The mice were allowed to acclimate for 2 to 3 days at 25°C before the transition.

### Open field test

Single-housed male mice (21 to 22 weeks old) with access to water and food were adapted for 30 min to a custom-made chamber (40 cm × 40 cm × 40 cm) in a ventilated soundproof booth. A camera was installed on the ceiling of the soundproof booths, and mice were allowed to move freely within the chamber for another 30 min. Light intensity was 120 to 140 lux. Light cycle experiment (3:00 to 4:00 PM) and dark cycle experiment (8:30 to 9:30 PM) were performed 4 days apart. A square-shaped area (20 cm × 20 cm) in the center was defined as the center zone, and the remaining area was defined as the outer zone. Locomotion was analyzed by EthoVision XT 15 (Noldus Wageningen, the Netherlands).

### Tissue staining

After the end of metabolic and behavioral experiments, all mice (22 to 23 weeks old) were deeply anesthetized with isoflurane and killed to harvest tissues. Liver, brown fat, epididymal fat, and inguinal fat tissues were isolated and fixed in neutralized formaldehyde solution (HT501128, Sigma). Paraffin-embedded tissue sections were stained with HE, or oil red O. Images were obtained with a slide scanner (Axio Scan. Z1, Carl Zeiss).

### Drugs

Tertiapin-Q (STT-170, Alomone Labs), CGP54626 (1088, Tocris), baclofen (0417, Tocris), linopirdine (1999, Tocris), XE991 (2000, Tocris), PK-THPP (5338, Tocris), spadin (5594, Tocris), and tolbutamide (T0891, dissolution with alcohol, Sigma) were used in whole-cell patch clamp mode. Kynurenic acid (K3375, Sigma), picrotoxin (1128, Tocris), and tetrodotoxin (T-550, Alomone Labs) were used to block synaptic currents. All solutions used in this study were made according to manufacturer’s specifications, and stock solutions of all drugs were dissolved in autoclaved de-ionized water unless specifically stated.

### Data analysis

Statistical analysis was done using GraphPad Prism 7 (GraphPad Software). Statistical data are expressed as mean ± SEM, where *n* represents the number of cells or mice studied. The significance of differences between groups was evaluated using two-tailed paired or unpaired Student’s *t* test, with a confidence level of *p* < 0.05 (*), *p* < 0.01 (**), or *p* < 0.001 (***). For some analyses, we used ordinary one-way ANOVA (to compare values between 3 experimental groups) or two-way repeated measures ANOVA (to compare time-dependent changes of values between groups) with a confidence level of *p* < 0.05 (*), *p* < 0.01 (**), *p* < 0.001 (***), or *p* < 0.0001 (****). We used the Bonferroni correction for all post hoc tests of ANOVA.

## Supporting information

S1 Fig Electrical properties of NPY neurons grouped by the response to tertiapin-Q. **Related to Fig 1.** Bar graphs and dots summarize action potential (AP) frequency (A), RMP (B), input resistance (C), and AP threshold (D) of cells depolarized by 100 nM, 300 nM, or 500 nM tertiapin-Q (Depol) vs. cells that did not respond (No Response). (A) AP frequency was 1.9 ± 0.5 Hz (*n* = 15) and 3.5 ± 0.3 Hz (*n* = 20) in “Depol” and “No Response” cells, respectively (df = 33, t = 2.798, *p* = 0.009). (B) RMP was −47.4 ± 2.2 mV (*n* = 15) and −41.2 ± 0.7 mV (*n* = 20) in “Depol” and “No Response” cells, respectively (df = 33, t = 3.045, *p* = 0.005). (C) Input resistance was 2.39 ± 0.22 GΩ (*n* = 15) and 2.89 ± 0.19 GΩ (*n* = 20) in “Depol” and “No Response” cells, respectively (df = 33, t = 1.739, *p* = 0.091). (D) AP threshold was −30.7 ± 0.7 mV (*n* = 12) and −28.3 ± 0.4 mV (*n* = 20) in “Depol” and “No Response” cells, respectively (df = 30, t = 3.128, *p* = 0.004). Data are presented as mean ± SEM. Unpaired *t* test was used for statistical analyses. **p < 0.01. The numerical data for S1A–S1D Fig can be found in S1 Data.
(TIF)

S2 Fig Effects of K^+^ channel blockers on RMP of NPY neurons. **Related to Fig 1.** (A) Trace demonstrates depolarizing effects of linopirdine and XE991, M channels blockers. (B) Trace demonstrates no effects of PK-THPP, a TASK-3 channel blocker. (C) Trace demonstrates no effects of spadin, a TREK-1 channel blocker. (D) Trace demonstrates no effects of tolbutamide, a K_ATP_ channel blocker. (E–H) Bar graphs and dots summarize effects on RMP change of linopirdine and XE991 (from −40.4 ± 0.7 mV to −39.5 ± 0.7 mV, *n* = 12, df = 11, t = 1.650, *p* = 0.127) (E), PK-THPP (from −42.5 ± 1.0 mV to −42.1 ± 0.8 mV, *n* = 12, df = 11, t = 0.890, *p* = 0.393) (F), spadin (from −41.9 ± 1.1 mV to −42.3 ± 1.0 mV, *n* = 13, df = 12, t = 1.866, *p* = 0.087) (G), and tolbutamide (from −42.2 ± 0.7 mV to −41.7 ± 0.8 mV, *n* = 13,df = 12, t = 1.879, and *p* = 0.085) (H). Red and black lines indicate changes of membrane potential in depolarized and nonresponsive neurons, respectively. Data are presented as mean ± SEM. Paired *t* test was used for statistical analyses. ns = not significant. The numerical data for S2E–S2H Fig can be found in S1 Data.
(TIF)

S3 Fig Expression of *Girk* mRNA by arcuate AgRP neurons. **Related to Fig 2.** (A) Graph demonstrates percentage of *Agrp* (+) neurons that express mRNA of *Girk1* and/or *Girk2*. *Girk1* (green): *Girk1*-containing *Agrp* (+) neurons; *Girk2* (magenta): *Girk2*-containing *Agrp* (+) neurons; *Girk1* and *Girk2* (gray): *Agrp* (+) neurons containing both *Girk1* and *Girk2*. *n* = 3. (B) Graph demonstrates percentage of *Agrp* (+) neurons that express mRNA of *Girk1* and/or *Girk3*. *Girk1* (green): *Girk1*-containing *Agrp* (+) neurons; *Girk3* (cyan): *Girk3*-containing *Agrp* (+) neurons; *Girk1* and *Girk3* (gray): *Agrp* (+) neurons containing both *Girk1* and *Girk3*. *n* = 3. (C) Graph demonstrates percentage of *Agrp* (+) neurons that express mRNA of *Girk1* and/or *Girk4*. *Girk1* (green): *Girk1*-containing *Agrp* (+) neurons; *Girk4* (orange): *Girk4*-containing *Agrp* (+) neurons; *Girk1* and *Girk4* (gray): *Agrp* (+) neurons containing both *Girk1* and *Girk4*. *n* = 3. (D) Graph demonstrates percentage of *Agrp* (+) neurons that express mRNA of *Girk2* and/or *Girk3*. *Girk2* (magenta): *Girk2*-containing *Agrp* (+) neurons; *Girk3* (cyan): *Girk3*-containing *Agrp* (+) neurons; and *Girk2* and *Girk3* (gray): *Agrp* (+) neurons containing both *Girk2* and *Girk3*. *n* = 3. Data are presented as mean ± SEM. Twelve hypothalamic slices from each mouse (from bregma −1.58 mm to −2.02 mm) were included for analyses. See text for specific values. The numerical data for S3A–S3D Fig can be found in S2 Data.
(TIF)

S4 Fig Role of GIRK2-containing GIRK channels in GABA_B_-activated K^+^ current recorded from NPY neurons. **Related to Fig 3.** (A) Image demonstrates outward currents by local application of 100 μM baclofen. Voltage ramp pulses (from −120 mV to −10 mV, 100 mV/s) were applied as indicated by arrows, a and b, to obtain current responses, *I*_a_ and *I*_b_. (B) Image demonstrates current–voltage (I-V) relationship of baclofen-activated currents (*I*_*Bac*_); *I*_*Bac*_ was calculated by subtracting current responses (*I*_b_- *I*_a_) obtained in (A). (C) Rectification index was calculated by obtaining the ratio of amplitudes at −120 mV (*I*_-120 mV_) and −60 mV (*I*_-60 mV_) in 12 NPY neurons. (D, E) Images demonstrate *I*_*Bac*_ recorded from NPY^G2WT^ (black) and NPY^G2KO^ (red) neurons using 10 μM (D) or 100 μM (E) baclofen. (F, G) Image summarizes normalized amplitudes of *I*_Bac_ recorded from NPY^G2WT^ (black) and NPY^G2KO^ (red) neurons using 10 μM baclofen (1.4 ± 0.1 pA/pF, *n* = 32, for NPY^G2WT^ and 1.4 ± 0.1 pA/pF, *n* = 23, for NPY^G2KO^, df = 53, t = 0.276, *p* = 0.783) (F) and 100 μM baclofen (1.8 ± 0.1 pA/pF, *n* = 53, for NPY^G2WT^ and 1.8 ± 0.2 pA/pF, *n* = 26, for NPY^G2KO^, df = 77, t = 0.021, and *p* = 0.984) (G). Data are presented as mean ± SEM. Unpaired *t* test was used for statistical analyses. ns = not significant. The numerical data for S4C, S4F, and S4G Fig can be found in S3 Data.
(TIF)

S5 Fig Effects of tertiapin-Q on NPY^G2KO^ neurons. **Related to Fig 3.** (A, B) Lines and dots summarize effects of tertiapin-Q (300 nM) on RMP (from −44.8 ± 1.8 mV to −44.4 ± 1.7 mV, *n* = 13, df = 12, t = 0.856, *p* = 0.409) (A) and input resistance (from 2.85 ± 0.24 GΩ to 2.80 ± 0.30 GΩ, *n* = 13, df = 12, t = 0.299, *p* = 0.770) (B) of NPY^G2KO^ neurons. Red and black lines indicate changes of membrane potential or input resistance in depolarized and nonresponsive neurons, respectively. (C) Bar graphs and dots summarize changes of membrane potentials by tertiapin-Q (300 nM) in NPY^G2WT^ neurons and NPY^G2KO^ neurons (2.8 ± 1.0 mV, *n* = 11, for NPY^G2WT^ and 0.4 ± 0.4 mV, *n* = 13, for NPY^G2KO^, df = 22, t = 2.354, *p* = 0.028). Data are presented as mean ± SEM. Paired *t* test (A and B) and unpaired *t* test (C) were used for statistical analyses. *p < 0.05, ns = not significant. The numerical data for S5A–S5C Fig can be found in S3 Data.
(TIF)

S6 Fig Effects of CGP54626 on NPY^G2WT^ neurons. **Related to Fig 3.** (A) Image demonstrates no effects of CGP54626 on NPY^G2WT^ neurons. Dotted line indicates RMP. (B) Lines and dots summarize effects of CGP54626 on RMP (from −42.9 ± 0.8 mV to −43.2 ± 0.8 mV, *n* = 12, df = 11, t = 2.191, *p* = 0.051). (C) Lines and dots summarize effect of CGP54626 on input resistance (from 2.68 ± 0.20 GΩ to 2.71 ± 0.21 GΩ, *n* = 12, df = 11, t = 0.519, *p* = 0.614). Paired *t* test was used for statistical analyses. ns = not significant. The numerical data for S6B and S6C Fig can be found in S3 Data.
(TIF)

S7 Fig Validation of AgRP neuron-specific *Girk1* and *Girk2* deletion. **Related to Fig 4.** (A, B) Left panels demonstrate mRNA of *Agrp* (magenta) and *Girk1* (cyan) detected by in situ hybridization (ISH) experiments within the arcuate nucleus of GIRK1^WT^ (A) and GIRK1^AgRP-KO^ mice (B). 3V = third ventricle. Scale bar = 50 μm. Right panels of (A) and (B) demonstrate magnified images of black rectangular area in the left panels of (A) and (B). *Girk1* (+) *Agrp-*expressing neurons are marked by red arrowheads, and *Girk1* (-) *Agrp*-expressing neurons are marked by black arrowheads. Scale bar = 10 μm. (C, D) Bar graphs and dots summarize the proportion of *Girk1*-expressing AgRP neurons (25.5 ± 2.8%, *n* = 3, for GIRK1^WT^ and 6.7 ± 2.0%, *n* = 3, for GIRK1^AgRP-KO^, df = 4, t = 5.489, *p* = 0.005) (C) and *Girk2*-expressing AgRP neurons (46.2 ± 4.3%, *n* = 3, for GIRK1^WT^ and 47.6 ± 3.0%, *n* = 3, for GIRK1^AgRP-KO^, df = 4, t = 0.248, *p* = 0.817) (D) in GIRK1^WT^ (*n* = 3) and GIRK1^AgRP-KO^ (*n* = 3) mice. (E, F) Left panels demonstrate mRNA of *Agrp* (magenta) and *Girk2* (cyan) detected by ISH experiments within the arcuate nucleus of GIRK2^WT^ (E) and GIRK2^AgRP-KO^ mice (F). 3V = third ventricle. Scale bar = 50 μm. Right panels of (E) and (F) demonstrate magnified images of black rectangular area in the left panels of (E) and (F). *Girk2* (+) *Agrp-*expressing neurons are marked by red arrowheads, and *Girk2* (-) *Agrp*-expressing neurons are marked by black arrowheads. Scale bar = 10 μm. (G, H) Bar graphs and dots summarize the proportion of *Girk2*-expressing AgRP neurons (57.1 ± 3.8%, *n* = 3, for GIRK2^WT^ and 13.2 ± 2.1%, *n* = 3, for GIRK2^AgRP-KO^, df = 4, t = 10.08, *p* = 0.0005) (G) and *Girk1*-expressing AgRP neurons (32.0 ± 5.1%, *n* = 3, for GIRK2^WT^ and 25.7 ± 4.1%, *n* = 3, for GIRK2^AgRP-KO^, df = 4, t = 0.972, *p* = 0.386) (H) in GIRK2^WT^ (*n* = 3) and GIRK2^AgRP-KO^ (*n* = 3) mice. A total of 16 hypothalamic slices from each mouse (from bregma −1.46 mm to −2.06 mm) were included for analyses. Data are presented as mean ± SEM. Unpaired *t* test was used for statistical analyses. **p < 0.01, ***p < 0.001, ns = not significant. The numerical data for S7C, S7D, S7G, and S7H Fig can be found in S4 Data.
(TIF)

S8 Fig Expression of *Girk1* and *Girk2* mRNA by hippocampal neurons. **Related to Fig 4.** (A, B) Images demonstrate *Girk1* mRNA (cyan) detected by ISH experiments in the hippocampus of GIRK1^WT^ (A) and GIRK1^AgRP-KO^ (B) mice. Scale bar = 200 μm. (C) Bar graphs and dots summarize normalized mRNA levels of *Girk1* by qRT-PCR of hippocampus in GIRK1^WT^ mice (WT, *n* = 5) and GIRK1^AgRP-KO^ mice (KO, *n* = 6) (1.01 ± 0.06, *n* = 5, for GIRK1^WT^ and 1.04 ± 0.08, *n* = 6, for GIRK1^AgRP-KO^, df = 9, t = 0.279, *p* = 0.787 in left graph; 1.00 ± 0.03, *n* = 5, for GIRK1^WT^ and 0.89 ± 0.15, *n* = 6, for GIRK1^AgRP-KO^, df = 9, t = 0.682, *p* = 0.513 in right graph). (D, E) Images demonstrate *Girk2* mRNA (cyan) detected by ISH experiments in the hippocampus of GIRK2^WT^ (D) and GIRK2^AgRP-KO^ (E) mice. Scale bar = 200 μm. (F) Bar graphs and dots summarize normalized mRNA levels of *Girk2* by qRT-PCR of hippocampus in GIRK2^WT^ mice (WT, *n* = 5) and GIRK2^AgRP-KO^ mice (KO, *n* = 6) (1.02 ± 0.11, *n* = 5, for GIRK2^WT^ and 1.05 ± 0.09, *n* = 6, for GIRK2^AgRP-KO^, df = 9, t = 0.190, *p* = 0.854 in left graph; 1.00 ± 0.04, *n* = 5, for GIRK2^WT^ and 0.87 ± 0.15, *n* = 6, for GIRK2^AgRP-KO^, df = 9, t = 0.819, and *p* = 0.434 in right graph). Data are presented as mean ± SEM. Unpaired *t* test was used for statistical analyses. ns = not significant. The numerical data for S8C and S8F Fig can be found in S4 Data.
(TIF)

S9 Fig Locomotion and anxiety-like behavior of GIRK2^WT^ and GIRK2^AgRP-KO^ mice. **Related to Fig 6.** (A) Bar graphs and dots summarize ambulatory movement of GIRK2^WT^ (*n* = 14) and GIRK2^AgRP-KO^ (*n* = 16) mice (260.8 ± 48.5 counts, *n* = 14, for GIRK2^WT^ and 256.9 ± 48.8 counts, *n* = 16, for GIRK2^AgRP-KO^, df = 28, t = 0.057, *p* = 0.955 in dark cycle; 58.0 ± 10.3 counts, *n* = 14, for GIRK2^WT^ and 51.6 ± 8.1 counts, *n* = 16, for GIRK2^AgRP-KO^, df = 28, t = 0.4921, *p* = 0.627 in light cycle). (B) Bar graphs and dots summarize rearing activity of GIRK2^WT^ (*n* = 14) and GIRK2^AgRP-KO^ (*n* = 16) mice (148.3 ± 27.1 counts, *n* = 14, for GIRK2^WT^ and 146.3 ± 32.0 counts, *n* = 16, for GIRK2^AgRP-KO^, df = 28, t = 0.049, *p* = 0.962 in dark cycle; 26.7 ± 9.6 counts, *n* = 14, for GIRK2^WT^ and 18.7 ± 3.9 counts, *n* = 16, for GIRK2^AgRP-KO^, df = 28, t = 0.804, *p* = 0.428 in light cycle). (C) Trajectory of freely moving GIRK2^WT^ (*n* = 8) and GIRK2^AgRP-KO^ (*n* = 9) mice in the OFT chamber in dark and light cycles. (D) Bar graphs and dots summarize total moving distance of GIRK2^WT^ (*n* = 8) and GIRK2^AgRP-KO^ (*n* = 9) mice (95.1 ± 9.0 m, *n* = 8, for GIRK2^WT^ and 108.1 ± 4.1 m, *n* = 9, for GIRK2^AgRP-KO^, df = 15, t = 1.370, *p* = 0.191 in dark cycle; 113.8 ± 6.6 m, *n* = 8, for GIRK2^WT^ and 123.9 ± 7.8 m, *n* = 9, for GIRK2^AgRP-KO^, df = 15, t = 0.980, *p* = 0.343 in light cycle). (E) Image demonstrates a view of chamber by a camera that is installed on the ceiling of sound-proof booths. (F) Heat-maps demonstrate zone preference of GIRK2^WT^ and GIRK2^AgRP-KO^ mice in the chamber. (G) Bar graphs and dots summarize proportions of duration in center and outer zones of GIRK2^WT^ (*n* = 8) and GIRK2^AgRP-KO^ (*n* = 9) mice (10.6 ± 1.8%, *n* = 8, for GIRK2^WT^ and 8.4 ± 0.6%, *n* = 9, for GIRK2^AgRP-KO^, df = 15, t = 1.224, *p* = 0.240 in dark cycle and center; 13.4 ± 1.4%, *n* = 8, for GIRK2^WT^ and 12.3 ± 1.6%, *n* = 9, for GIRK2^AgRP-KO^, df = 15, t = 0.523, *p* = 0.609 in light cycle and center; 89.4 ± 1.8%, *n* = 8, for GIRK2^WT^ and 91.6 ± 0.6%, *n* = 9, for GIRK2^AgRP-KO^, df = 15, t = 1.224, *p* = 0.240 in dark cycle and outer; 86.6 ± 1.4%, *n* = 8, for GIRK2^WT^ and 87.8 ± 1.6%, *n* = 9, for GIRK2^AgRP-KO^, df = 15, t = 0.523, *p* = 0.609 in light cycle and outer). Data are presented as mean ± SEM. Unpaired *t* test was used for statistical analyses. ns = not significant. The numerical data for S9A, S9B, S9D, and S9G Fig can be found in S6 Data.
(TIF)

S10 Fig Effects of ghrelin on food intake. **Related to Fig 7.** Graph demonstrates food intake of GIRK2^WT^ mice (black, *n* = 4) and GIRK2^AgRP-KO^ mice (red, *n* = 4) after i.p. injections of saline (filled circles) or ghrelin (0.4 mg/kg, empty circles). Mice were injected at 10 AM and food intake was measured for the next 4 h. Data are presented as mean ± SEM. Two-way repeated measures ANOVA with Bonferroni correction was used for statistical analyses. Group (df = 3, *F*_*3*, *12*_ = 12.03, *p* = 0.0006), time (df = 4, *F*_*4*, *48*_ = 23.99, *p* < 0.0001), interaction (df = 12, *F*_*12*, *48*_ = 4.83, *p* < 0.0001). *p < 0.05; **, ##*p* < 0.01; ****, ####*p* < 0.0001. ns = not significant. Saline, GIRK2^WT^ vs. Ghrelin, GIRK2^WT^ (*). Saline, GIRK2^AgRP-KO^ vs. Ghrelin, GIRK2^AgRP-KO^ (#). Saline, GIRK2^WT^ vs. Saline, GIRK2^AgRP-KO^ (ns). Ghrelin, GIRK2^WT^ vs. Ghrelin, GIRK2^AgRP-KO^ (ns). The numerical data for S10 Fig can be found in S7 Data.
(TIF)

S1 Data Original data for the graphs in Figs 1 and S1 and S2. Each tab includes data for individual panels of Figs 1 and S1 and S2.
(XLSX)

S2 Data Original data for the graphs in Figs 2 and S3. Each tab includes data for individual panels of Figs 2 and S3.
(XLSX)

S3 Data Original data for the graphs in Figs 3 and S4–S6. Each tab includes data for individual panels of Figs 3 and S4–S6.
(XLSX)

S4 Data Original data for the graphs in Figs 4 and S7 and S8. Each tab includes data for individual panels of Figs 4 and S7 and S8.
(XLSX)

S5 Data Original data for the graphs in Fig 5. Each tab includes data for individual panels of Fig 5.
(XLSX)

S6 Data Original data for the graphs in Figs 6 and S9. Each tab includes data for individual panels of Figs 6 and S9.
(XLSX)

S7 Data Original data for the graphs in Figs 7 and S10. Each tab includes data for individual panels of Figs 7 and S10.
(XLSX)

S8 Data Original data for Table 1. Numbers represent individual numerical data for changes of membrane potential and reversal potential in Table 1.
(XLSX)

## Abbreviations

AgRP: agouti-related peptide
AP: action potential
ARH: arcuate nucleus of the hypothalamus
BAT: brown adipose tissue
cDNA: complimentary DNA
ChAT: choline acetyltransferase
EE: energy expenditure
FISH: fluorescence in situ hybridization
GIRK: G protein-gated inwardly rectifying K^+^
HE: hematoxylin and eosin
HRP: horseradish peroxidase
IACUC: Institutional Animal Care and Use Committee
IGW: inguinal white
IHC: immunohistochemistry
IML: intermediolateral column; i.p., intraperitoneally
ISH: in situ hybridization
KAIST: Korea Advanced Institute of Science and Technology
NCD: normal chow diet
NDS: normal donkey serum
NMR: nuclear magnetic resonance
NPY: neuropeptide Y
OFT: open field test
PCR: polymerase chain reaction
PGW: perigonadal white
POMC: pro-opiomelanocortin
RMP: resting membrane potential
RT: room temperature
TTX: tetrodotoxin
UCP-1: uncoupling protein-1
