# Supplementary figures and images for: GIRK2 potassium channels expressed by the AgRP neurons decrease adiposity and body weight in mice
Source: PLoS Biol. 2023 Aug 18;21(8):e3002252. doi: 10.1371/journal.pbio.3002252 (PMC10468093; doi:10.1371/journal.pbio.3002252)

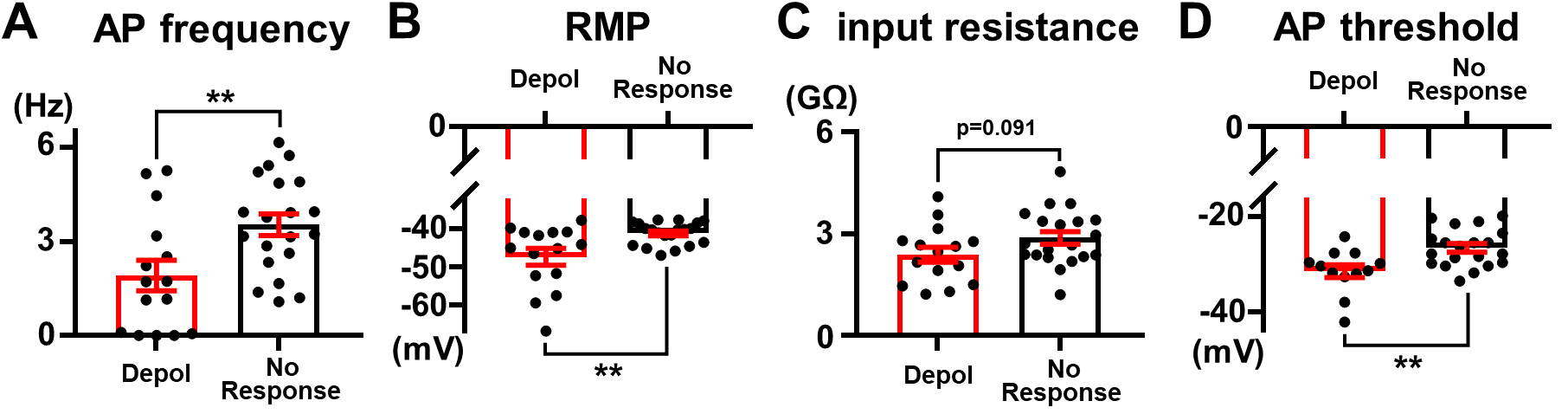

Supplement: S1 Fig — Related to Fig 1. Bar graphs and dots summarize action potential (AP) frequency (A), RMP (B), input resistance (C), and AP threshold (D) of cells depolarized by 100 nM, 300 nM, or 500 nM tertiapin-Q (Depol) vs. cells that did not respond (No Response). (A) AP frequency was 1.9 ± 0.5 Hz (n = 15) and 3.5 ± 0.3 Hz (n = 20) in “Depol” and “No Response” cells, respectively (df = 33, t = 2.798, p = 0.009). (B) RMP was −47.4 ± 2.2 mV (n = 15) and −41.2 ± 0.7 mV (n = 20) in “Depol” and “No Response” cells, respectively (df = 33, t = 3.045, p = 0.005). (C) Input resistance was 2.39 ± 0.22 GΩ (n = 15) and 2.89 ± 0.19 GΩ (n = 20) in “Depol” and “No Response” cells, respectively (df = 33, t = 1.739, p = 0.091). (D) AP threshold was −30.7 ± 0.7 mV (n = 12) and −28.3 ± 0.4 mV (n = 20) in “Depol” and “No Response” cells, respectively (df = 30, t = 3.128, p = 0.004). Data are presented as mean ± SEM. Unpaired t test was used for statistical analyses. **p < 0.01. The numerical data for S1A–S1D Fig can be found in S1 Data. (TIF) [file pbio.3002252.s001.tif]

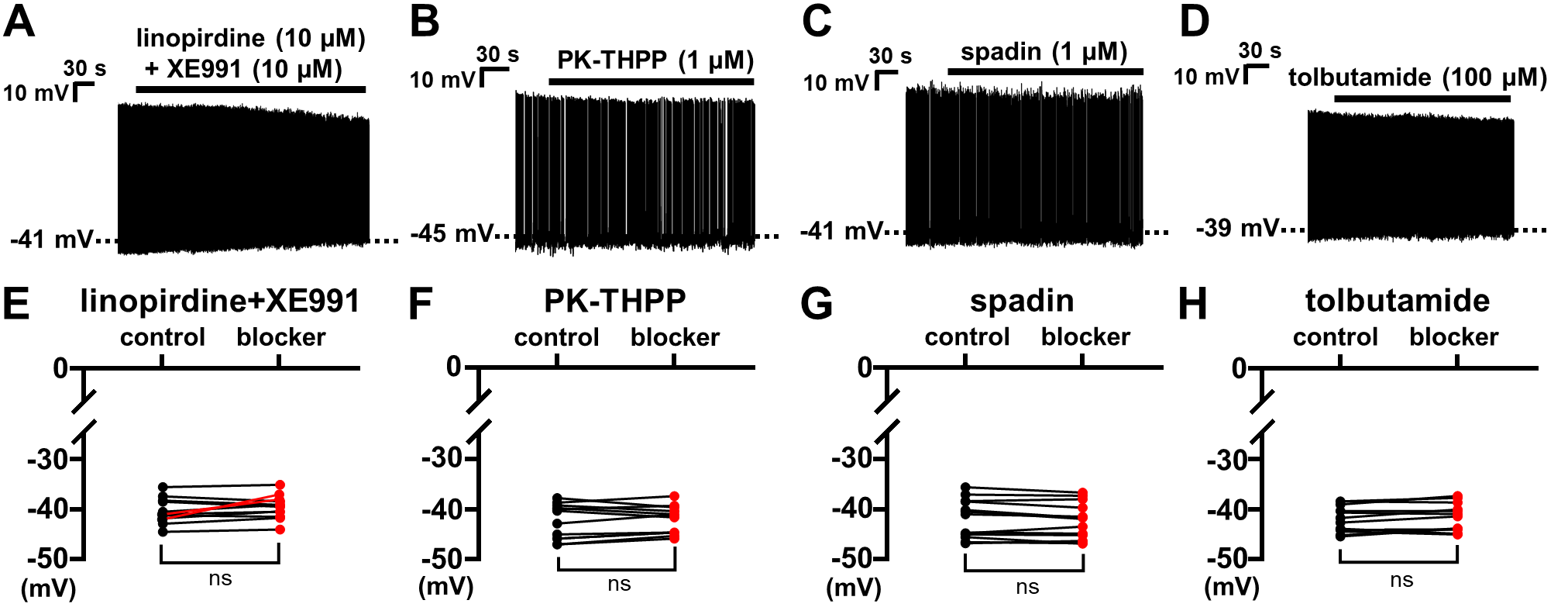

Supplement: S2 Fig — Related to Fig 1. (A) Trace demonstrates depolarizing effects of linopirdine and XE991, M channels blockers. (B) Trace demonstrates no effects of PK-THPP, a TASK-3 channel blocker. (C) Trace demonstrates no effects of spadin, a TREK-1 channel blocker. (D) Trace demonstrates no effects of tolbutamide, a KATP channel blocker. (E–H) Bar graphs and dots summarize effects on RMP change of linopirdine and XE991 (from −40.4 ± 0.7 mV to −39.5 ± 0.7 mV, n = 12, df = 11, t = 1.650, p = 0.127) (E), PK-THPP (from −42.5 ± 1.0 mV to −42.1 ± 0.8 mV, n = 12, df = 11, t = 0.890, p = 0.393) (F), spadin (from −41.9 ± 1.1 mV to −42.3 ± 1.0 mV, n = 13, df = 12, t = 1.866, p = 0.087) (G), and tolbutamide (from −42.2 ± 0.7 mV to −41.7 ± 0.8 mV, n = 13,df = 12, t = 1.879, and p = 0.085) (H). Red and black lines indicate changes of membrane potential in depolarized and nonresponsive neurons, respectively. Data are presented as mean ± SEM. Paired t test was used for statistical analyses. ns = not significant. The numerical data for S2E–S2H Fig can be found in S1 Data. (TIF) [file pbio.3002252.s002.tif]

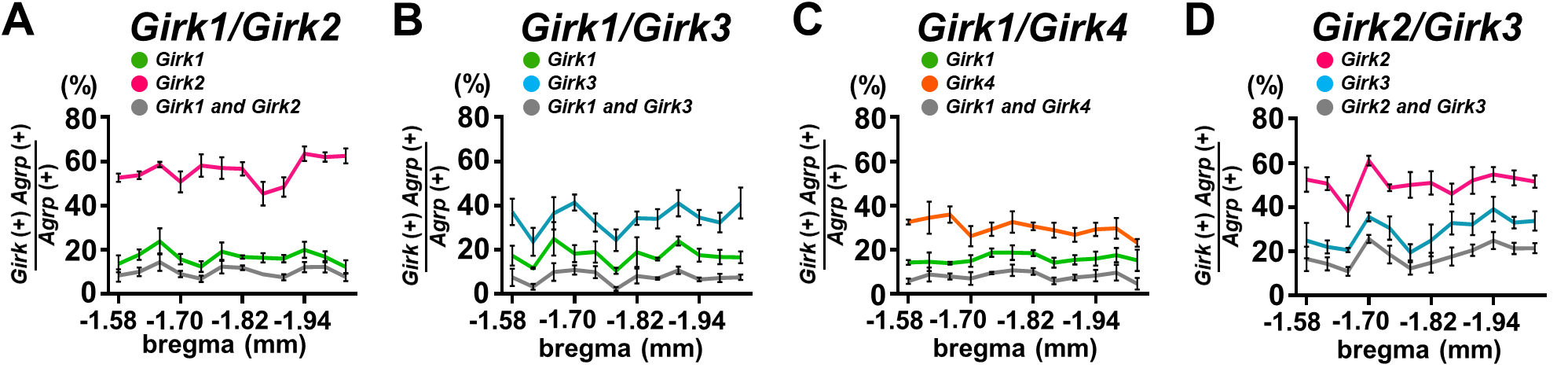

Supplement: S3 Fig — Related to Fig 2. (A) Graph demonstrates percentage of Agrp (+) neurons that express mRNA of Girk1 and/or Girk2. Girk1 (green): Girk1-containing Agrp (+) neurons; Girk2 (magenta): Girk2-containing Agrp (+) neurons; Girk1 and Girk2 (gray): Agrp (+) neurons containing both Girk1 and Girk2. n = 3. (B) Graph demonstrates percentage of Agrp (+) neurons that express mRNA of Girk1 and/or Girk3. Girk1 (green): Girk1-containing Agrp (+) neurons; Girk3 (cyan): Girk3-containing Agrp (+) neurons; Girk1 and Girk3 (gray): Agrp (+) neurons containing both Girk1 and Girk3. n = 3. (C) Graph demonstrates percentage of Agrp (+) neurons that express mRNA of Girk1 and/or Girk4. Girk1 (green): Girk1-containing Agrp (+) neurons; Girk4 (orange): Girk4-containing Agrp (+) neurons; Girk1 and Girk4 (gray): Agrp (+) neurons containing both Girk1 and Girk4. n = 3. (D) Graph demonstrates percentage of Agrp (+) neurons that express mRNA of Girk2 and/or Girk3. Girk2 (magenta): Girk2-containing Agrp (+) neurons; Girk3 (cyan): Girk3-containing Agrp (+) neurons; and Girk2 and Girk3 (gray): Agrp (+) neurons containing both Girk2 and Girk3. n = 3. Data are presented as mean ± SEM. Twelve hypothalamic slices from each mouse (from bregma −1.58 mm to −2.02 mm) were included for analyses. See text for specific values. The numerical data for S3A–S3D Fig can be found in S2 Data. (TIF) [file pbio.3002252.s003.tif]

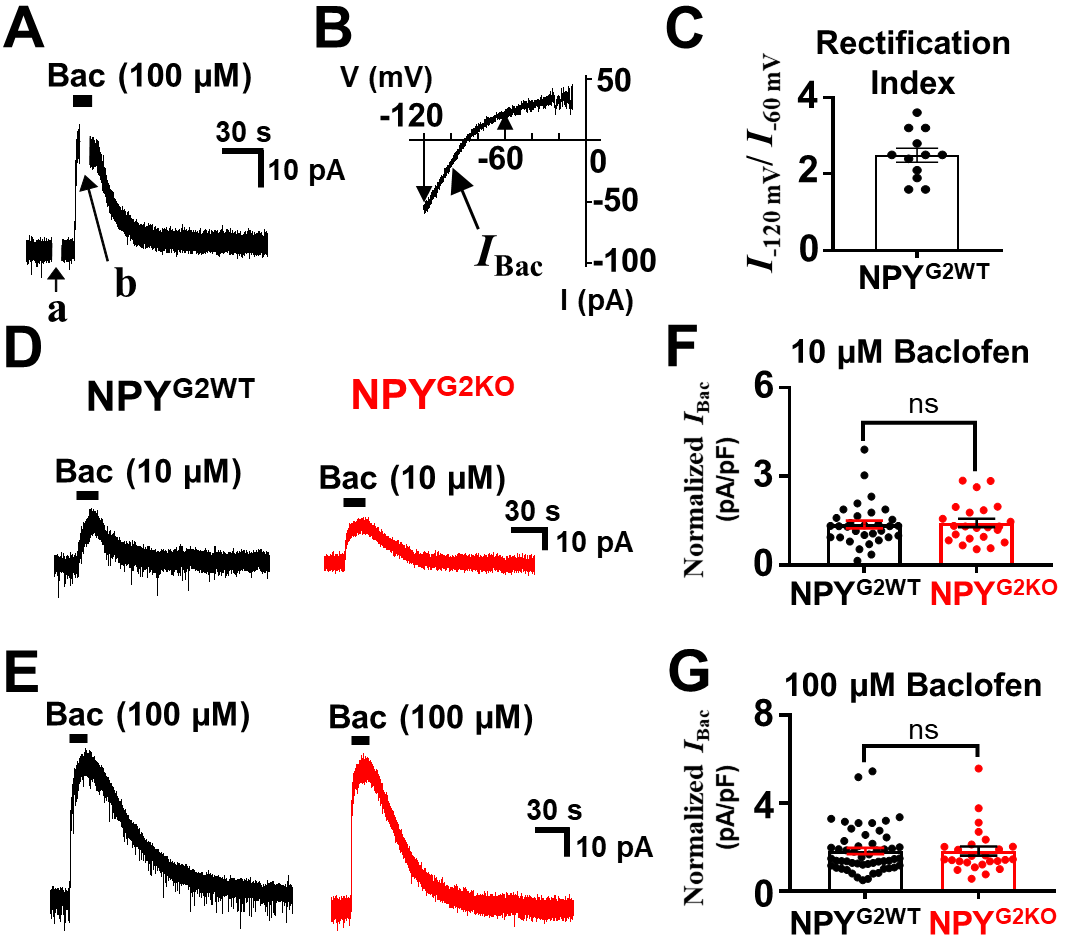

Supplement: S4 Fig — Related to Fig 3. (A) Image demonstrates outward currents by local application of 100 μM baclofen. Voltage ramp pulses (from −120 mV to −10 mV, 100 mV/s) were applied as indicated by arrows, a and b, to obtain current responses, Ia and Ib. (B) Image demonstrates current–voltage (I-V) relationship of baclofen-activated currents (IBac); IBac was calculated by subtracting current responses (Ib- Ia) obtained in (A). (C) Rectification index was calculated by obtaining the ratio of amplitudes at −120 mV (I-120 mV) and −60 mV (I-60 mV) in 12 NPY neurons. (D, E) Images demonstrate IBac recorded from NPYG2WT (black) and NPYG2KO (red) neurons using 10 μM (D) or 100 μM (E) baclofen. (F, G) Image summarizes normalized amplitudes of IBac recorded from NPYG2WT (black) and NPYG2KO (red) neurons using 10 μM baclofen (1.4 ± 0.1 pA/pF, n = 32, for NPYG2WT and 1.4 ± 0.1 pA/pF, n = 23, for NPYG2KO, df = 53, t = 0.276, p = 0.783) (F) and 100 μM baclofen (1.8 ± 0.1 pA/pF, n = 53, for NPYG2WT and 1.8 ± 0.2 pA/pF, n = 26, for NPYG2KO, df = 77, t = 0.021, and p = 0.984) (G). Data are presented as mean ± SEM. Unpaired t test was used for statistical analyses. ns = not significant. The numerical data for S4C, S4F, and S4G Fig can be found in S3 Data. (TIF) [file pbio.3002252.s004.tif]

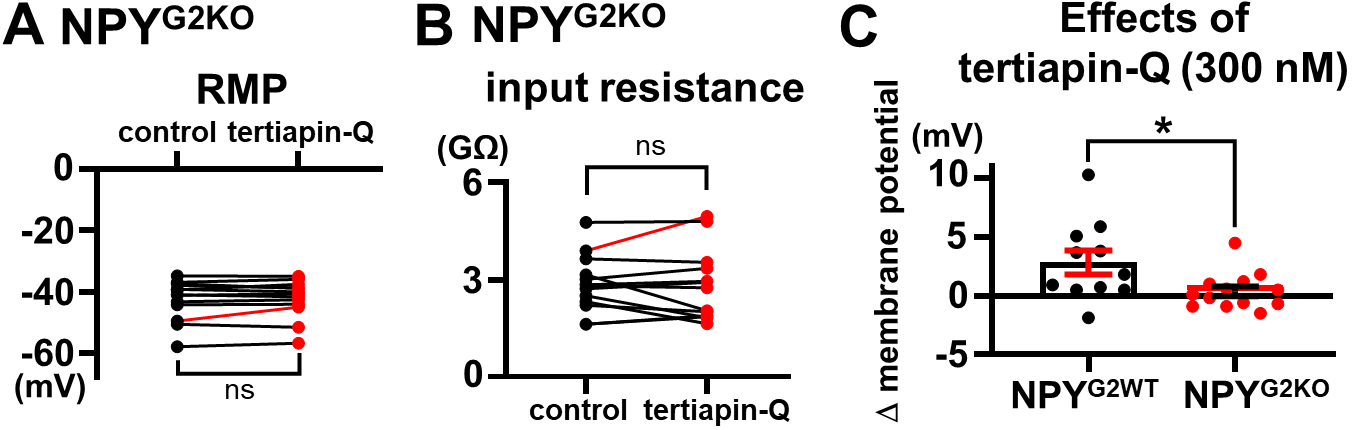

Supplement: S5 Fig — Related to Fig 3. (A, B) Lines and dots summarize effects of tertiapin-Q (300 nM) on RMP (from −44.8 ± 1.8 mV to −44.4 ± 1.7 mV, n = 13, df = 12, t = 0.856, p = 0.409) (A) and input resistance (from 2.85 ± 0.24 GΩ to 2.80 ± 0.30 GΩ, n = 13, df = 12, t = 0.299, p = 0.770) (B) of NPYG2KO neurons. Red and black lines indicate changes of membrane potential or input resistance in depolarized and nonresponsive neurons, respectively. (C) Bar graphs and dots summarize changes of membrane potentials by tertiapin-Q (300 nM) in NPYG2WT neurons and NPYG2KO neurons (2.8 ± 1.0 mV, n = 11, for NPYG2WT and 0.4 ± 0.4 mV, n = 13, for NPYG2KO, df = 22, t = 2.354, p = 0.028). Data are presented as mean ± SEM. Paired t test (A and B) and unpaired t test (C) were used for statistical analyses. *p < 0.05, ns = not significant. The numerical data for S5A–S5C Fig can be found in S3 Data. (TIF) [file pbio.3002252.s005.tif]

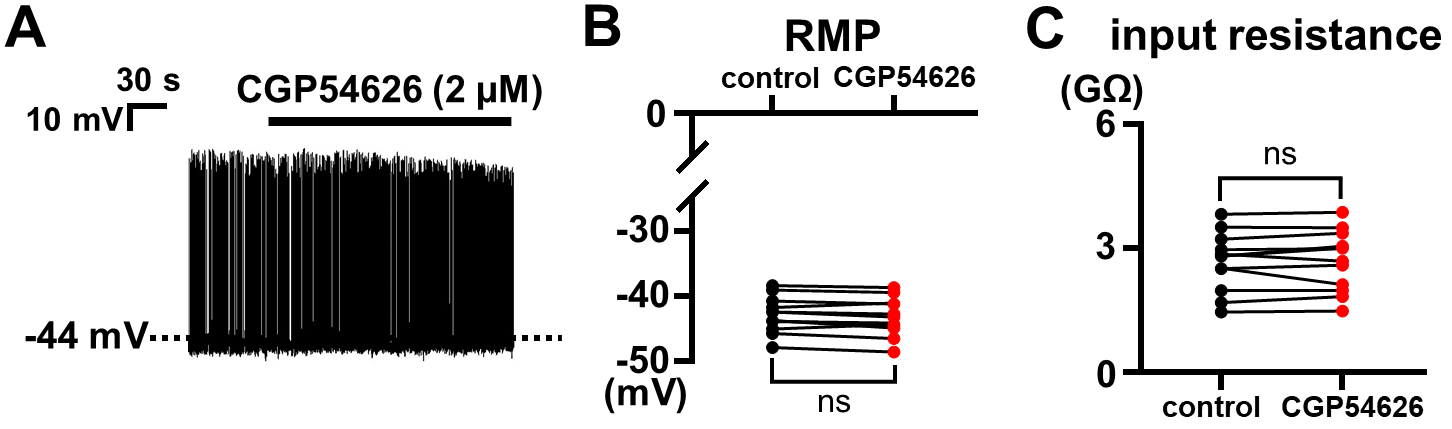

Supplement: S6 Fig — Related to Fig 3. (A) Image demonstrates no effects of CGP54626 on NPYG2WT neurons. Dotted line indicates RMP. (B) Lines and dots summarize effects of CGP54626 on RMP (from −42.9 ± 0.8 mV to −43.2 ± 0.8 mV, n = 12, df = 11, t = 2.191, p = 0.051). (C) Lines and dots summarize effect of CGP54626 on input resistance (from 2.68 ± 0.20 GΩ to 2.71 ± 0.21 GΩ, n = 12, df = 11, t = 0.519, p = 0.614). Paired t test was used for statistical analyses. ns = not significant. The numerical data for S6B and S6C Fig can be found in S3 Data. (TIF) [file pbio.3002252.s006.tif]

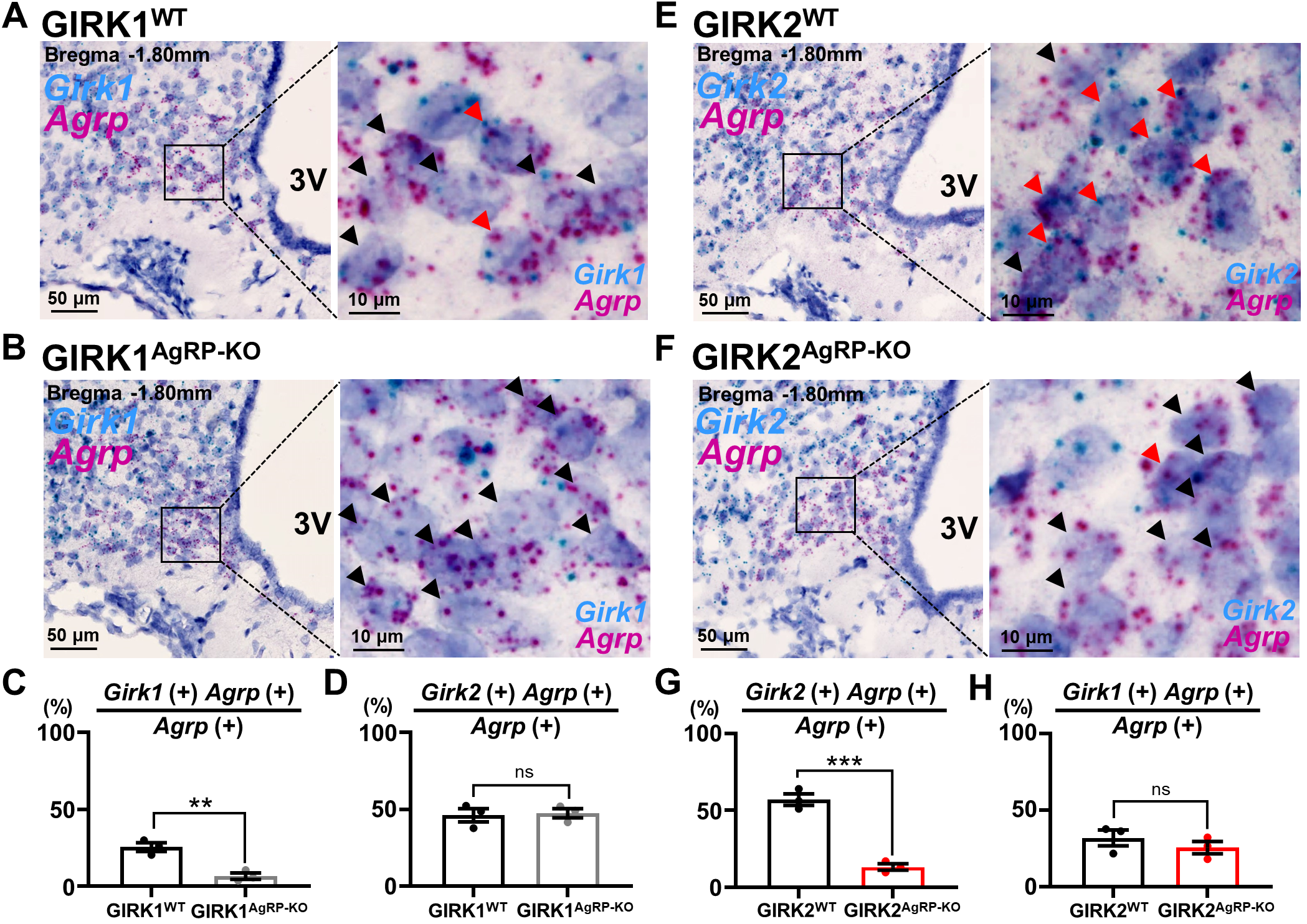

Supplement: S7 Fig — Related to Fig 4. (A, B) Left panels demonstrate mRNA of Agrp (magenta) and Girk1 (cyan) detected by in situ hybridization (ISH) experiments within the arcuate nucleus of GIRK1WT (A) and GIRK1AgRP-KO mice (B). 3V = third ventricle. Scale bar = 50 μm. Right panels of (A) and (B) demonstrate magnified images of black rectangular area in the left panels of (A) and (B). Girk1 (+) Agrp-expressing neurons are marked by red arrowheads, and Girk1 (-) Agrp-expressing neurons are marked by black arrowheads. Scale bar = 10 μm. (C, D) Bar graphs and dots summarize the proportion of Girk1-expressing AgRP neurons (25.5 ± 2.8%, n = 3, for GIRK1WT and 6.7 ± 2.0%, n = 3, for GIRK1AgRP-KO, df = 4, t = 5.489, p = 0.005) (C) and Girk2-expressing AgRP neurons (46.2 ± 4.3%, n = 3, for GIRK1WT and 47.6 ± 3.0%, n = 3, for GIRK1AgRP-KO, df = 4, t = 0.248, p = 0.817) (D) in GIRK1WT (n = 3) and GIRK1AgRP-KO (n = 3) mice. (E, F) Left panels demonstrate mRNA of Agrp (magenta) and Girk2 (cyan) detected by ISH experiments within the arcuate nucleus of GIRK2WT (E) and GIRK2AgRP-KO mice (F). 3V = third ventricle. Scale bar = 50 μm. Right panels of (E) and (F) demonstrate magnified images of black rectangular area in the left panels of (E) and (F). Girk2 (+) Agrp-expressing neurons are marked by red arrowheads, and Girk2 (-) Agrp-expressing neurons are marked by black arrowheads. Scale bar = 10 μm. (G, H) Bar graphs and dots summarize the proportion of Girk2-expressing AgRP neurons (57.1 ± 3.8%, n = 3, for GIRK2WT and 13.2 ± 2.1%, n = 3, for GIRK2AgRP-KO, df = 4, t = 10.08, p = 0.0005) (G) and Girk1-expressing AgRP neurons (32.0 ± 5.1%, n = 3, for GIRK2WT and 25.7 ± 4.1%, n = 3, for GIRK2AgRP-KO, df = 4, t = 0.972, p = 0.386) (H) in GIRK2WT (n = 3) and GIRK2AgRP-KO (n = 3) mice. A total of 16 hypothalamic slices from each mouse (from bregma −1.46 mm to −2.06 mm) were included for analyses. Data are presented as mean ± SEM. Unpaired t test was used for statistical analyses. **p < 0.01, ***p < 0.001, [file pbio.3002252.s007.tif]

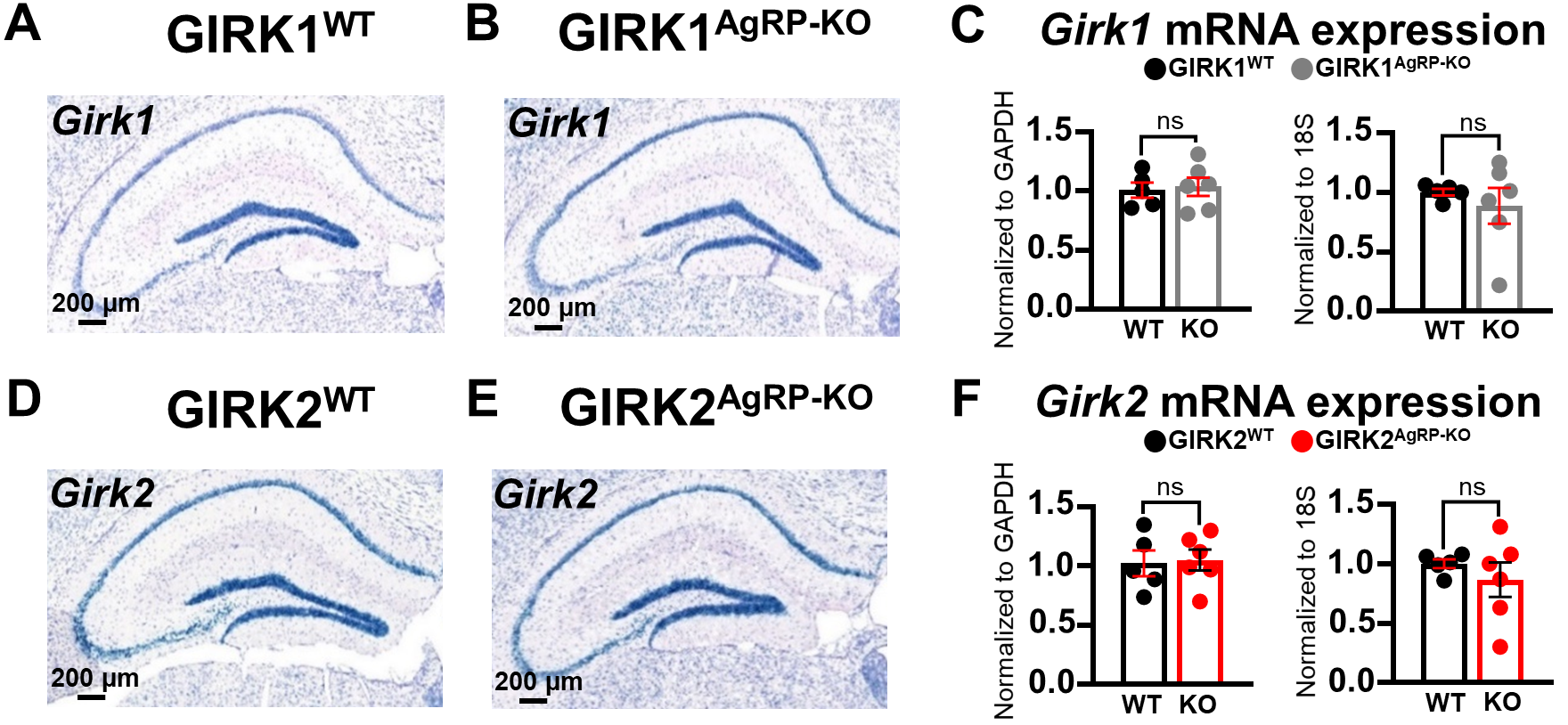

Supplement: S8 Fig — Related to Fig 4. (A, B) Images demonstrate Girk1 mRNA (cyan) detected by ISH experiments in the hippocampus of GIRK1WT (A) and GIRK1AgRP-KO (B) mice. Scale bar = 200 μm. (C) Bar graphs and dots summarize normalized mRNA levels of Girk1 by qRT-PCR of hippocampus in GIRK1WT mice (WT, n = 5) and GIRK1AgRP-KO mice (KO, n = 6) (1.01 ± 0.06, n = 5, for GIRK1WT and 1.04 ± 0.08, n = 6, for GIRK1AgRP-KO, df = 9, t = 0.279, p = 0.787 in left graph; 1.00 ± 0.03, n = 5, for GIRK1WT and 0.89 ± 0.15, n = 6, for GIRK1AgRP-KO, df = 9, t = 0.682, p = 0.513 in right graph). (D, E) Images demonstrate Girk2 mRNA (cyan) detected by ISH experiments in the hippocampus of GIRK2WT (D) and GIRK2AgRP-KO (E) mice. Scale bar = 200 μm. (F) Bar graphs and dots summarize normalized mRNA levels of Girk2 by qRT-PCR of hippocampus in GIRK2WT mice (WT, n = 5) and GIRK2AgRP-KO mice (KO, n = 6) (1.02 ± 0.11, n = 5, for GIRK2WT and 1.05 ± 0.09, n = 6, for GIRK2AgRP-KO, df = 9, t = 0.190, p = 0.854 in left graph; 1.00 ± 0.04, n = 5, for GIRK2WT and 0.87 ± 0.15, n = 6, for GIRK2AgRP-KO, df = 9, t = 0.819, and p = 0.434 in right graph). Data are presented as mean ± SEM. Unpaired t test was used for statistical analyses. ns = not significant. The numerical data for S8C and S8F Fig can be found in S4 Data. (TIF) [file pbio.3002252.s008.tif]

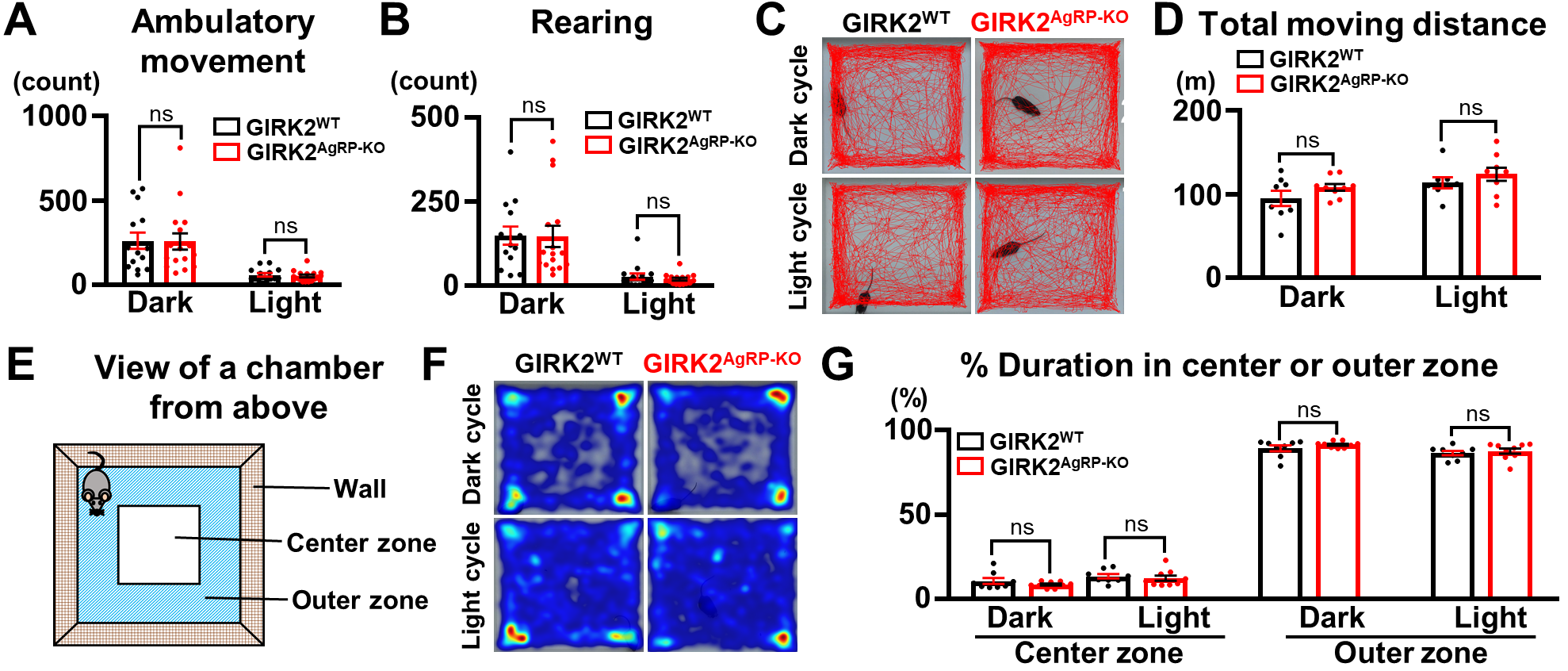

Supplement: S9 Fig — Related to Fig 6. (A) Bar graphs and dots summarize ambulatory movement of GIRK2WT (n = 14) and GIRK2AgRP-KO (n = 16) mice (260.8 ± 48.5 counts, n = 14, for GIRK2WT and 256.9 ± 48.8 counts, n = 16, for GIRK2AgRP-KO, df = 28, t = 0.057, p = 0.955 in dark cycle; 58.0 ± 10.3 counts, n = 14, for GIRK2WT and 51.6 ± 8.1 counts, n = 16, for GIRK2AgRP-KO, df = 28, t = 0.4921, p = 0.627 in light cycle). (B) Bar graphs and dots summarize rearing activity of GIRK2WT (n = 14) and GIRK2AgRP-KO (n = 16) mice (148.3 ± 27.1 counts, n = 14, for GIRK2WT and 146.3 ± 32.0 counts, n = 16, for GIRK2AgRP-KO, df = 28, t = 0.049, p = 0.962 in dark cycle; 26.7 ± 9.6 counts, n = 14, for GIRK2WT and 18.7 ± 3.9 counts, n = 16, for GIRK2AgRP-KO, df = 28, t = 0.804, p = 0.428 in light cycle). (C) Trajectory of freely moving GIRK2WT (n = 8) and GIRK2AgRP-KO (n = 9) mice in the OFT chamber in dark and light cycles. (D) Bar graphs and dots summarize total moving distance of GIRK2WT (n = 8) and GIRK2AgRP-KO (n = 9) mice (95.1 ± 9.0 m, n = 8, for GIRK2WT and 108.1 ± 4.1 m, n = 9, for GIRK2AgRP-KO, df = 15, t = 1.370, p = 0.191 in dark cycle; 113.8 ± 6.6 m, n = 8, for GIRK2WT and 123.9 ± 7.8 m, n = 9, for GIRK2AgRP-KO, df = 15, t = 0.980, p = 0.343 in light cycle). (E) Image demonstrates a view of chamber by a camera that is installed on the ceiling of sound-proof booths. (F) Heat-maps demonstrate zone preference of GIRK2WT and GIRK2AgRP-KO mice in the chamber. (G) Bar graphs and dots summarize proportions of duration in center and outer zones of GIRK2WT (n = 8) and GIRK2AgRP-KO (n = 9) mice (10.6 ± 1.8%, n = 8, for GIRK2WT and 8.4 ± 0.6%, n = 9, for GIRK2AgRP-KO, df = 15, t = 1.224, p = 0.240 in dark cycle and center; 13.4 ± 1.4%, n = 8, for GIRK2WT and 12.3 ± 1.6%, n = 9, for GIRK2AgRP-KO, df = 15, t = 0.523, p = 0.609 in light cycle and center; 89.4 ± 1.8%, n = 8, for GIRK2WT and 91.6 ± 0.6%, n = 9, for GIRK2AgRP-KO, df = 15, t = 1.224, p = 0.240 in dark cycle and outer; 86.6 ± 1.4%, n = 8, for GIRK [file pbio.3002252.s009.tif]

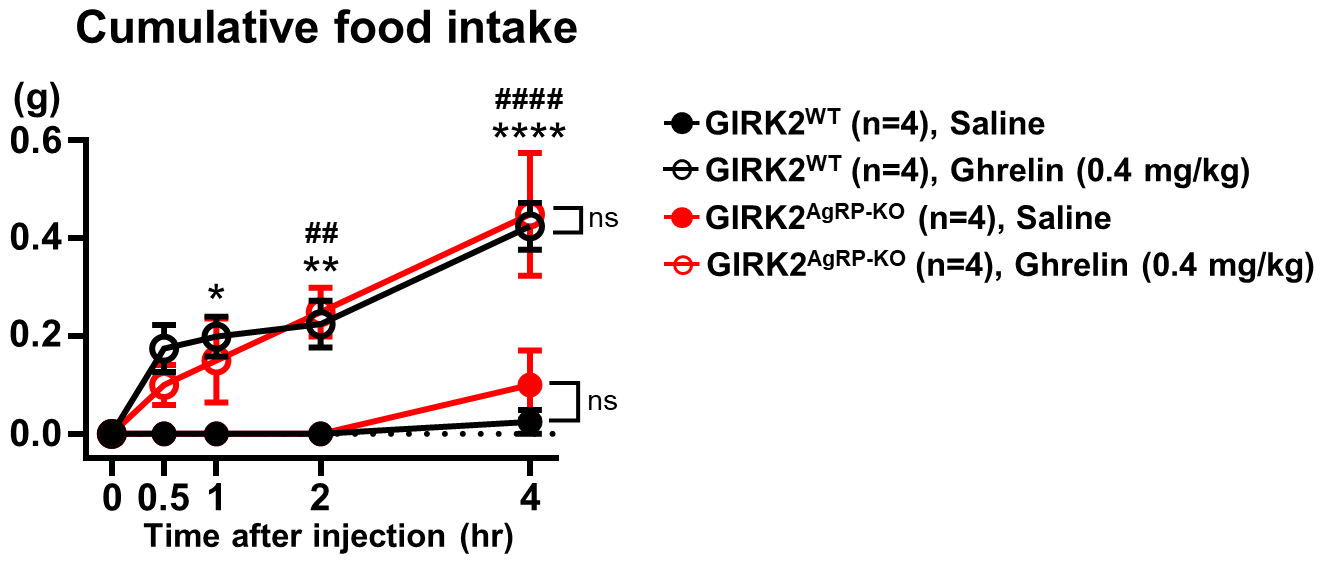

Supplement: S10 Fig — Related to Fig 7. Graph demonstrates food intake of GIRK2WT mice (black, n = 4) and GIRK2AgRP-KO mice (red, n = 4) after i.p. injections of saline (filled circles) or ghrelin (0.4 mg/kg, empty circles). Mice were injected at 10 AM and food intake was measured for the next 4 h. Data are presented as mean ± SEM. Two-way repeated measures ANOVA with Bonferroni correction was used for statistical analyses. Group (df = 3, F3, 12 = 12.03, p = 0.0006), time (df = 4, F4, 48 = 23.99, p < 0.0001), interaction (df = 12, F12, 48 = 4.83, p < 0.0001). *p < 0.05; **, ##p < 0.01; ****, ####p < 0.0001. ns = not significant. Saline, GIRK2WT vs. Ghrelin, GIRK2WT (*). Saline, GIRK2AgRP-KO vs. Ghrelin, GIRK2AgRP-KO (#). Saline, GIRK2WT vs. Saline, GIRK2AgRP-KO (ns). Ghrelin, GIRK2WT vs. Ghrelin, GIRK2AgRP-KO (ns). The numerical data for S10 Fig can be found in S7 Data. (TIF) [file pbio.3002252.s010.tif]
